# Low-dose *i*nterleukin 2 for the reduction of *v*ascular inflammati*o*n in acute corona*ry* syndromes (IVORY): protocol and study rationale for a randomised, double-blind, placebo-controlled, phase II clinical trial

**DOI:** 10.1136/bmjopen-2022-062602

**Published:** 2022-10-07

**Authors:** Rouchelle Sriranjan, Tian Xiao Zhao, Jason Tarkin, Annette Hubsch, Joanna Helmy, Evangelia Vamvaka, Navazh Jalaludeen, Simon Bond, Stephen P Hoole, Philip Knott, Samantha Buckenham, Victoria Warnes, Nick Bird, Heok Cheow, Heike Templin, Paul Cacciottolo, James H F Rudd, Ziad Mallat, Joseph Cheriyan

**Affiliations:** 1 Department of Medicine, Division of Cardiovascular Medicine, University of Cambridge, Cambridge, UK; 2 Department of Medicine, Division of Experimental Medicine and Immunotherapeutics (EMIT), University of Cambridge, Cambridge, UK; 3 Cambridge Clinical Trials Unit, Cambridge University Hospitals NHS Foundation Trust, Cambridge, UK; 4 Cardiology, Papworth Hospital NHS Foundation Trust, Cambridge, UK; 5 Department of Clinical Immunology, Cambridge University Hospitals NHS Foundation Trust, Cambridge, UK; 6 Department of Nuclear Medicine, Cambridge University Hospitals NHS Foundation Trust, Cambridge, UK

**Keywords:** cardiology, cardiovascular imaging, immunology, clinical trials

## Abstract

**Introduction:**

Inflammation plays a critical role in the pathogenesis of atherosclerosis, the leading cause of ischaemic heart disease (IHD). Studies in preclinical models have demonstrated that an increase in regulatory T cells (Tregs), which have a potent immune modulatory action, led to a regression of atherosclerosis. The Low-dose InterLeukin 2 (IL-2) in patients with stable ischaemic heart disease and Acute Coronary Syndromes (LILACS) study, established the safety of low-dose IL-2 and its biological efficacy in IHD. The IVORY trial is designed to assess the effects of low-dose IL-2 on vascular inflammation in patients with acute coronary syndromes (ACS).

**Methods and analysis:**

In this study, we hypothesise that low-dose IL-2 will reduce vascular inflammation in patients presenting with ACS. This is a double-blind, randomised, placebo-controlled, phase II clinical trial. Patients will be recruited across two centres, a district general hospital and a tertiary cardiac centre in Cambridge, UK. Sixty patients with ACS (unstable angina, non-ST elevation myocardial infarction or ST elevation myocardial infarction) with high-sensitivity C reactive protein (hsCRP) levels >2 mg/L will be randomised to receive either 1.5×10^6^ IU of low-dose IL-2 or placebo (1:1). Dosing will commence within 14 days of admission. Dosing will comprise of an induction and a maintenance phase. 2-Deoxy-2-[fluorine-18] fluoro-D-glucose (^18^F-FDG) positron emission tomography/CT (PET/CT) scans will be performed before and after dosing. The primary endpoint is the change in mean maximum target to background ratios (TBR_max_) in the index vessel between baseline and follow-up scans. Changes in circulating T-cell subsets will be measured as secondary endpoints of the study. The safety and tolerability of extended dosing with low-dose IL-2 in patients with ACS will be evaluated throughout the study.

**Ethics and dissemination:**

The Health Research Authority and Health and Care Research Wales, UK (19/YH/0171), approved the study. Written informed consent is required to participate in the trial. The results will be reported through peer-reviewed journals and conference presentations.

**Trial registration number:**

NCT04241601.

**Strengths and limitations of this study**

- This study explores the clinical efficacy of low-dose interleukin 2 (IL-2) in reducing vascular inflammation in patients acutely presenting with acute coronary syndrome (ACS), using a well-validated imaging biomarker (^18^F-FDG (2-deoxy-2-[fluorine-18] fluoro-D-glucose)).
- Changes that occur in a wide range of circulating lymphocyte subsets with extended dosing with low-dose IL-2 will be studied in the acutely presenting ACS patient population at multiple timepoints.
- The safety of extended dosing with low-dose IL-2 in patients acutely presenting with ACS will be studied in this trial, using multiple sources.
- Due to the early phase nature of this study, it is not powered to determine if treatment with low-dose IL-2 would lead to improvements in clinical outcomes.

## Introduction

### Atherosclerosis and inflammation

Inflammation plays a pivotal role in the initiation and progression of atherosclerosis, which accounts for an overwhelming majority of cardiovascular events. The role of the immune system in the pathophysiology of atherosclerosis and plaque instability is multifaceted and orchestrated by both the innate and adaptive components of the immune system.^1^ The Canakinumab ANti-inflammatory Thrombosis Outcomes Study reported for the first time that anti-inflammatory therapy significantly reduced recurrent cardiovascular events in patients with ischaemic heart disease (IHD) and ongoing inflammation with persistently raised high-sensitivity C reactive protein (hsCRP) levels.^2^ Further efforts to harness the immune system for pharmacological therapeutic strategies in cardiovascular disease are currently underway.^3 4^

Due to their critical role in mediating immune tolerance and antiatherosclerotic effects, natural and inducible regulatory T cells (Tregs) (CD4^+^CD25^+^FOPX3) are a focus of novel therapeutic strategies. Initial studies in preclinical models showed that Tregs reduced plaque inflammation, slowed progression and even led to the regression of atherosclerosis.^5 6^ Tregs have also been implicated in moderating the postischaemic immune responses triggered by self-antigens presented by necrotic myocardium.^7^ In line with this finding, it has also been demonstrated that Tregs promote myocardial wound healing, attenuate adverse left ventricular remodelling and improve cardiac function.^8–11^ The Malmo Diet and Cancer study further demonstrated an increased risk of acute coronary events in patients with low levels of baseline circulating Treg cells.^12^ Observational studies have also shown that patients with acute coronary syndrome (ACS) have lower numbers of circulating Tregs with impaired function.^13–16^ Furthermore, low levels of Tregs maybe also associated with plaque instability.^15^

Interleukin 2 (IL-2) plays a crucial role in immune homeostasis by determining the balance between T effector (Teffs) and Tregs.^17 18^ While IL-2 is implicated in the development of both Teffs and Tregs, unlike Teffs, Treg cells constitutively express an IL-2 receptor (IL-2Rα) which binds to IL-2 with high affinity, making this subset very sensitive to IL-2.^19^ Of the cytokines involved in Treg development and stability, IL-2 is the most essential.^20^

The administration of low-dose IL-2 has been used in autoimmune conditions such as graft versus host disease, autoimmune vasculitis, hepatitis, type 1 diabetes mellitus and systemic lupus erythematosus, to boost Treg numbers with promising results.^17 21–24^ Furthermore, a recent study involving patients with 11 autoimmune conditions demonstrated the clinical efficacy and safety of low-dose IL-2.^25^

## LILACS

The Low-dose InterLeukin 2 in patients with stable ischaemic heart disease and Acute Coronary Syndromes (LILACS) was the first study to explore the safety of low dose of IL-2 and its ability to expand Treg cells in this patient cohort. This was a double-blinded, randomised controlled adaptive phase I/IIa trial.^26^ Twenty-five patients with stable IHD were dosed in part A with aldesleukin (recombinant IL-2). The dose range used in this trial was between 0.3×10^6^ and 3×10^6^ IU. The ratio of IL-2 to placebo was 3:2 for each group. An increase in Treg cells was observed at the doses tested without any significant increases in Teff cells.^26^ Part B, which included patients with ACS (non-ST elevation myocardial infarction (NSTEMI) and unstable angina (UA)), was carried out on completion of part A. Two doses (1.5×10^6^ and 2.5×10^6^ IU) were used in part B (IL-2: placebo=6:2). In both parts, the low-dose IL-2 and placebo were administered for 5 consecutive days. Low-dose IL-2 was well tolerated in both patients with stable IHD and ACS. The most commonly occurring adverse reactions were injection site reactions, which were temporary. Other adverse reactions included influenza-like symptoms and myalgia. No serious adverse events (SAEs) were observed in part A. Two SAEs were noted in part B that were deemed to be unrelated to the investigational medical product (IMP). In LILACS, the target dose needed to achieve a 75% increase in Tregs was determined as 1.46×10^6^ IU.

Based on the promising results of LILACS, we hypothesised that low-dose IL-2 would reduce vascular inflammation in patients with ACS as assessed by 2-deoxy-2-[fluorine-18] fluoro-D-glucose (^18^F-FDG) positron emission tomography (PET)/CT imaging before and after treatment.

^18^F-FDG is the most widely used radionuclide in imaging inflammation in the context of atherosclerosis. Limitations of ^18^F-FDG include the fact that it cannot be used to quantify inflammation in coronary arteries reliably and that the ^18^F-FDG signal is not inflammatory cell specific as all glucose-metabolising cells take up ^18^F-FDG.^27–29^ However, with respect to atherosclerosis, ex vivo histological studies from arterial specimens have shown a strong correlation between ^18^F-FDG uptake and macrophage density.^30 31^ This is thought to be due to the increased glucose turnover in macrophages compared with other cells within the atherosclerotic plaque. Furthermore, ^18^F-FDG quantification is highly reliable and reproducible in large- to medium-sized vessels.^32^ ^18^F-FDG PET/CT has been used by our group and others as a biomarker for drug development in phase II cardiovascular drug intervention studies.^33–35^ ^18^F-FDG PET/CT has specifically been used to study the effect of statins and anti-inflammatory compounds on vascular inflammation.^36–40^

## Methods and analysis

The IVORY trial is a prospective, randomised, double-blind, placebo-controlled, phase II clinical trial. This is an academic study involving two centres namely, Cambridge University Hospitals (CUH) and Royal Papworth Hospital in Cambridge, UK. Overall study coordination, monitoring and auditing will be undertaken by the Cardiovascular Trials Office of the Cambridge Clinical Trials Unit (CCTU), CUH. The study is sponsored by CUH.

### Study population

To be included, participants must be admitted with a diagnosis of ACS. The study will include patients presenting with ST elevation myocardial infarction (STEMI), NSTEMI or UA. The participants should have symptoms suggestive of myocardial ischaemia lasting 10 min or longer at rest or on minimal effort and either have elevated levels of troponin on admission and/or dynamic ECG changes suggestive of ischaemia. Furthermore, to be included participants will require hsCRP levels >2 mg/L at screening. We hypothesise patients presenting with hsCRP levels >2 mg/L have residual systemic inflammation and are at higher risk of recurrent cardiovascular events. Therefore, recruitment will be restricted to these patients as they are most likely to benefit from this treatment.

Due to the ionising radiation associated with ^18^F-FDG PET/CT scans, to be included in the trial, women must be either postmenopausal, have had a documented hysterectomy and/or bilateral oophorectomy or sterilisation procedure or be perimenopausal with a negative pregnancy test at screening. Perimenopausal women will also have to comply with the use of contraception for the duration of the trial and undergo additional pregnancy tests during and after treatment.

Exclusion criteria include patients who present with cardiogenic shock, cardiac arrest and uncontrolled hypertension/hypotension. Blood tests will be carried out to exclude patients with thyroid, severe renal and hepatic dysfunction as specified in the protocol. Patients with active infection, history of malignancy requiring active treatment or on oral/intravenous immune suppression therapy will also be excluded. Patients with type 1, or type 2 diabetes mellitus with poor control, will be excluded due to the challenges in analysing ^18^F-FDG PET/CT scans in this patient cohort.^40^ Due to the ongoing pandemic and to minimise potential risk, patients who are COVID-19 positive on PCR at the time of screening will not be recruited to the trial. Detailed inclusion and exclusion criteria are listed in online supplemental table 1.

10.1136/bmjopen-2022-062602.supp2 Supplementary data

We plan to recruit 60 patients for the IVORY study. The sample size is based on an absolute difference of 0.2 in the primary endpoint (mean maximum target to background ratio (TBR_max_) in the index vessel) between placebo and active treatment at the end of the dosing period. This is equivalent to a 10% difference from a reference value of 2.02 and equivalent to the size effect observed after atheroprotective therapy.^35–37^ This is based on the results of previous studies that have reported less than a 10% difference in mean TBR_max_ in the index vessel between active treatment and placebo and failed to reduce cardiovascular outcomes.^34 39 41–43^

Assuming an SD of 0.24, 24 patients per arm, testing at two-sided 5% significance level, would provide 80% power. Therefore, a sample size of 30 completed patients per arm was selected to account for participant dropout and poor-quality scans deemed to be unsuitable for analysis (eg, because of motion artefact). This sample size of n=30 per group also allows the detection of a baseline-corrected 6% difference in TBRmax in the index vessel for the trial primary endpoint (mean 0.125, SD 0.166) between placebo and active treatment at the end of the treatment period, at 2-sided 5% significance level and 80% power.

The study opened on 26 August 2020. Thirty-three participants have completed the trial to date, and we expect recruitment to be completed by December 2023, after which the data analysis will commence.

### Dose selection for IVORY

A dose of 1.5×10^6^ IU was selected for the study based on the analysis of data from the LILACS trial. In patients with ACS dosed in Part B of the LILACS trial, a dose of 1.5×10^6^ IU led to an 80% increase in Tregs after 5 consecutive days of treatment. No significant increases in Teff cells were noted at this dose.

#### Active drug description

Aldesleukin is produced by recombinant DNA technology using an *Escherichia coli* strain that contains a genetically engineered modification of the human IL-2 gene.

Commercially available aldesleukin with an UK marketing authorisation will be used and initially prepared as per the manufacturer’s guidance. This preparation will be diluted further using 5% glucose, to give a final concentration of 3.3×10^6^ IU/mL. The administration of aldesleukin will be by subcutaneous injection.

#### Placebo description

Commercially available dextrose 5% with an UK marketing authorisation at equivalent dose volume will be used for the placebo formulation.

### Study protocol

The trial will be performed in accordance with the spirit and the letter of the Declaration of Helsinki, the conditions and principles of Good Clinical Practice, the protocol and applicable local regulatory requirements and law. Participants will be provided with an information sheet and given the opportunity to consider the information for at least 24 hours. They will have an opportunity during this time to discuss the study with trial staff and have any queries answered before consenting to participate in the trial. Full informed consent (online supplemental file) will be taken at the screening visit (V1) by the study team. The screening visit will also include baseline blood tests, a physical examination, vitals (blood pressure, heart rate, temperature), a clinical history and ECGs in triplicate. The screening bloods will include safety blood tests (full blood count, electrolytes/urea/creatinine, liver function tests), thyroid function tests, hsCRP, serum/urine pregnancy test where applicable and a full lipid profile.

10.1136/bmjopen-2022-062602.supp1 Supplementary data

Each participant who passes screening will be assigned an ID number. The research team will complete a case record form (CRF) for each encounter with the participant. Data are then sent to the CCTU for entry into the electronic trial database. All CRFs will be stored safely, and patient identifiable information is kept confidential.

Randomisation will then be carried out using sealed envelope (www.sealedenvelope.com). Randomisation will be stratified by ST elevation status to minimise bias between the groups since patients with STEMI typically have higher circulating hsCRP levels.

During the trial, the patient facing trial team, patients and data analysis teams (immunology sample analysers and image analysers) are blinded to the treatment allocation. To maintain the overall quality and legitimacy of the clinical trial, unblinding will only occur in exceptional circumstances when knowledge of the actual treatment is essential for further clinical management of the patient.

**Figure 1:**
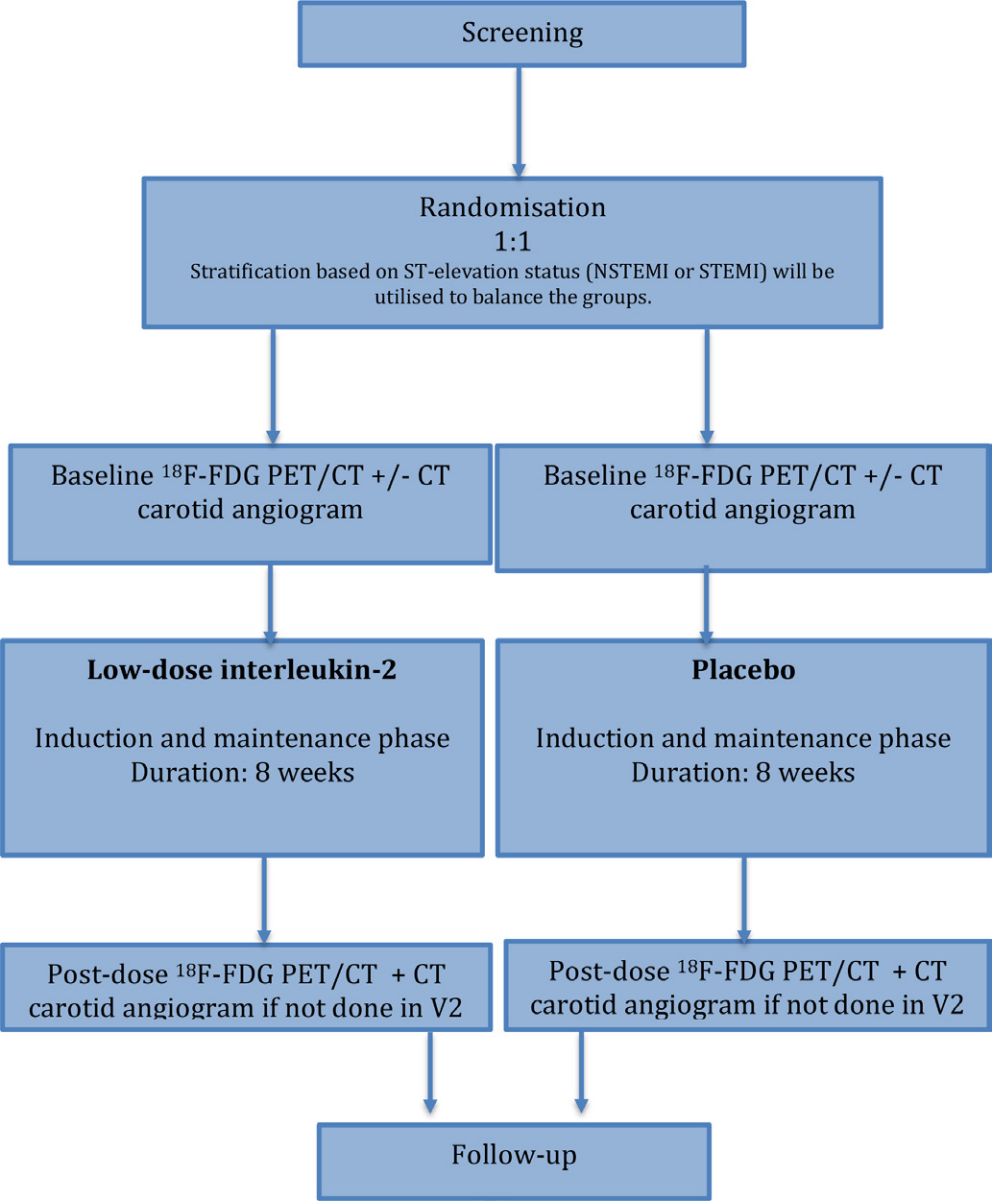
Summary of trial design. ^18^F-FDG, 2-deoxy-2-[fluorine-18] fluoro-D-glucose; NSTEMI, non-ST elevation myocardial infarction; PET, positron emission tomography; STEMI, ST elevation myocardial infarction.

The trial design is summarised in figure 1. After randomisation, patients will undergo an ^18^F-FDG PET/CT scan covering the ascending aorta and carotid arteries (V2).

The ^18^F-FDG PET/CT scans will be carried out in CUH. Patients would be instructed to fast for 6 hours prior to scanning. A finger prick blood sugar test will be performed prior to the PET/CT scan. A carotid angiogram will be undertaken after the PET/CT scan where possible. The carotid angiogram can be performed either in visit 2 or 15, as this is for anatomical correlation to enable more accurate measurement of ^18^F-FDG uptake in the carotid arteries. A transthoracic echocardiogram will also be undertaken prior to dosing to assess the left ventricular function and regional wall motion abnormalities.

**Figure 2:**
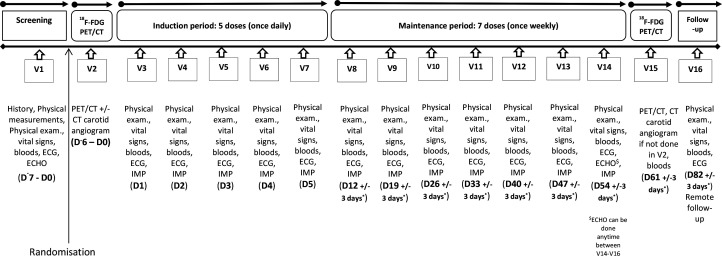
IVORY trial visits—timeline and assessments. ECHO, echocardiogram; ^18^F-FDG, 2-deoxy-2-[fluorine-18] fluoro-D-glucose; IMP, investigational medical product; PET, positron emission tomography.

Dosing will commence within 14 days of admission, and after the baseline ^18^F-FDG PET/CT scan (figure 2). Dosing will consist of two phases spanning a period of 8 weeks. The induction phase will involve once daily subcutaneous injections of IMP for 5 days (V3–V7). During the maintenance phase, participants will receive once weekly subcutaneous injections of IMP for 7 weeks. Dosing visits will last up to 1 hour during the induction phase. At each visit, participants will be screened for potential side effects and their medications will be reviewed by a clinician. Participants will also undergo a physical examination, have their vital signs measured, have ECGs recorded and safety bloods taken. Circulating systemic inflammatory biomarkers (hsCRP, interleukin 6), cardiac troponin I and lymphocyte subsets will be measured on V3 and V7.

During the maintenance phase (V8–V14), participants will be dosed once weekly. These visits will last for 1–1.5 hours. They will be similar to the induction phase visits; however, administration of the IMP will only occur once safety bloods taken during the visit are checked. Cardiac biomarkers and lymphocyte subsets will be measured during alternate maintenance visits (V8, V10, V12, V14). Within approximately 1 week from the completion of dosing, a second postdose ^18^F-FDG PET/CT scan will be undertaken. The final encounter will take place approximately 4 weeks after the final dosing visit via telephone unless an in-person visit is necessary for clinical or safety reasons.

Patients will be withdrawn from the trial after recruitment if any of the prespecified general, cardiac, renal or liver withdrawal criteria are met. They will also be withdrawn from the trial if they are positive for COVID-19 during dosing. The withdrawal criteria are summarised in table 1.

**Table 1:** IVORY study withdrawal criteria

General	Cardiorespiratory arrest. Failure to attend two scheduled dosing appointments without adequate reason based on the PI assessment. Severe hypersensitivity reactions will preclude any further IMP administration. New seizure activity/coma. Severe lethargy or somnolence. Respiratory insufficiency requiring intubation. Pregnancy. Withdrawal of consent. PI discretion. Any serious adverse reaction (AR) or adverse reaction which is deemed by investigators to be severe AR. Any significant incidental finding on PET/CT scan clinical governance reports, which in the opinion of the PI, necessitates further investigation and management. Any medical history, clinically relevant abnormality or reason that is deemed by the PI to make the patient ineligible to continue the trial.
Cardiac	In patients without left bundle branch block (LBBB) QTc interval >500 ms OR change from baseline: QTcB >60 ms. In patients with LBBB if baseline corrected QT interval increases from <450 ms to >500 ms OR from 450 to 480 ms to >530 ms. (withdrawal of patients is to be based on an average QTc value of triplicate ECGs.) Patients who will be scheduled to undergo coronary artery bypass grafting. Patients who develop new-onset severe pulmonary oedema/new severe congestive heart failure, requiring high dose >240 mg over 24 hours intravenous furosemide during admission. Patients with symptomatic uncontrolled systolic BP <80 mm Hg and/or diastolic BP <50 mm Hg (after at least two repeat recordings), or severe hypertension (as defined by BP >180 mm Hg systolic BP or >120 mm Hg diastolic BP on at least two readings). Patients with sustained (>30 s) or symptomatic ventricular tachycardia and patients with ventricular fibrillation will be withdrawn.
Renal	Serum creatinine elevation >2× baseline screening visit (V1).
Liver	ALT ≥3× ULN and total bilirubin ≥2× ULN. ALT ≥5× ULN. ALT ≥3× ULN if associated with symptoms (new or worsening) believed to be related to hepatitis (such as fatigue, nausea, vomiting, right upper quadrant pain or tenderness or jaundice) or hypersensitivity (such as fever, rash or eosinophilia). Isolated ALT ≥3× ULN that persists for ≥4 weeks.

ALT, alanine transaminase; BP, blood pressure; IMP, investigational medical product; PI, principal investigator; ULN, upper limit normal.

### Study procedures

#### ^18^F-FDG scans

A General Electric Discovery 690 PET/CT scanner (Milwaukee, Wisconsin, USA) or equivalent scanner will be used for imaging. This will be performed using reproducible, validated methods for image acquisition, reconstruction and interpretation as recommended by the European Association of Nuclear Medicine for the conduct of clinical trials using ^18^F-FDG PET/CT.^40^ A dose of approximately 240 Mbq ^18^F-FDG will be injected through a peripheral venous cannula. The ascending aorta will be imaged 90 min after the ^18^F-FDG injection. Attenuation correction and non-contrast CT scans of the ascending aorta will initially be performed. This will be followed by a single-bed PET scan acquired in 3D list mode for 10 min, with the superior portion of the aortic arch as the upper anatomical landmark of the scan. Carotid artery imaging will be undertaken immediately after the PET/CT scans of the ascending aorta. A single-bed PET scan will be acquired in 3D list mode for 15 min, with the external auditory meatus as the upper anatomical landmark of the scan. PET data for the ascending aorta and carotids will be reconstructed using iterative 3D time-of-flight-ordered subset expectation maximisation with standard corrections applied±point-spread function modelling to reduce partial volume error. A CT carotid angiogram will also be carried out after the PET acquisition either in visit 2 or visit 15.

All scans will be anonymised. The anonymised scans will be analysed in batches of 20 by the research team. Second person checks will also be performed on a subset of these scans.

#### Lymphocyte subset assays

Lymphocyte subset assays will be performed at the Department of Clinical Immunology, CUH, and this will occur within 4 hours of sample collection. The clinical investigators and laboratory technicians will be blinded to treatment allocation.

**Figure 3:**
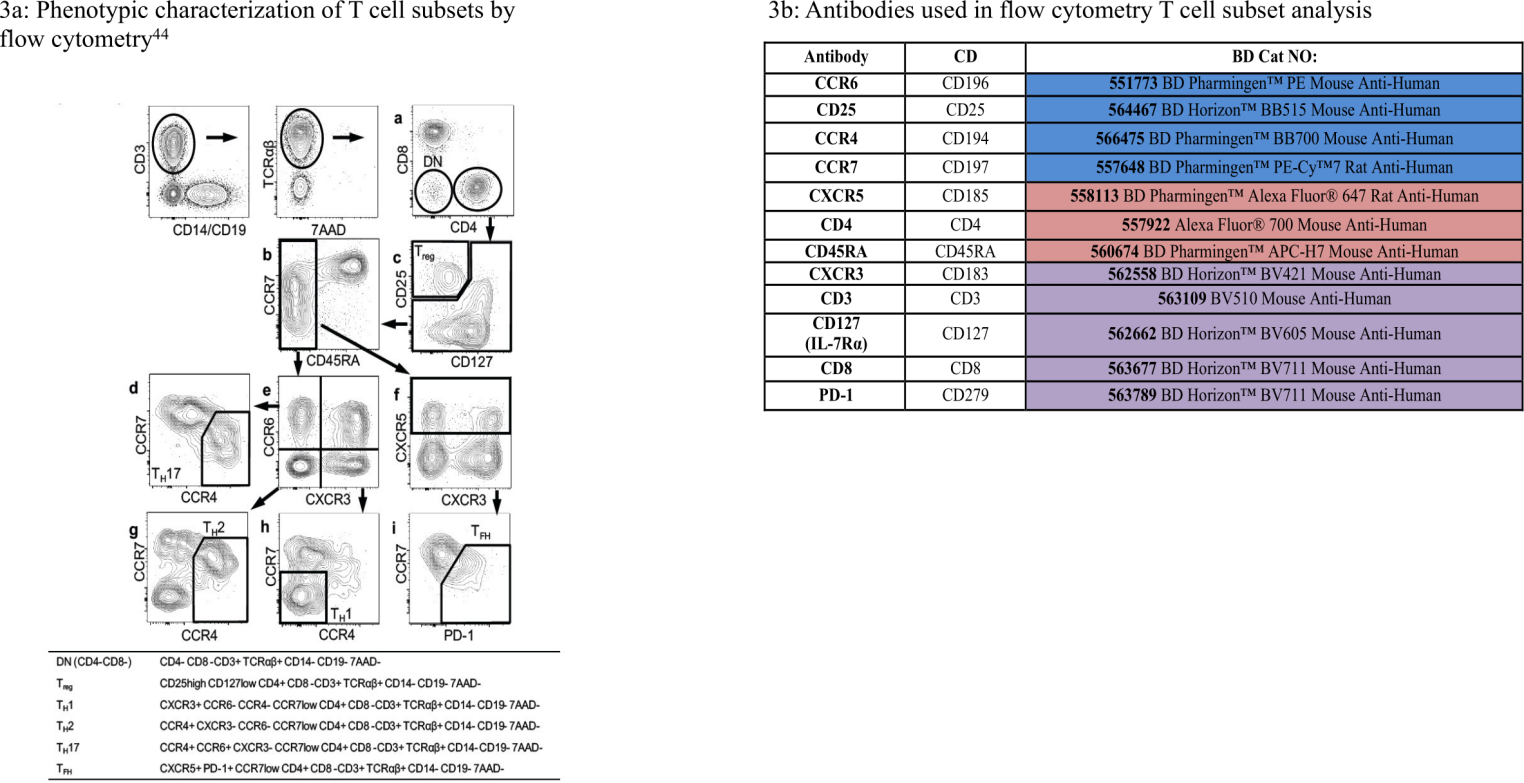
Lymphocyte subset analysis: A - Phenotypic characterisation of T cell subsets by flow cytometry; B -Antibodies used in fluorescence-activated cell sorting (FACS) analysis of T-cell subsets.

Whole blood will be assessed by performing fluorescence-activated cell sorting (FACS) to measure the absolute and percentage values for lymphocyte subsets (Teffs, T helper 1, T helper 2, T helper 17 and T follicular helper cells) and Treg cells. Antibody reagent of 50 µL and 50 µL whole blood will be added directly to the BD Trucount tube. The lyophilized pellet in the tube dissolves, releasing a known number of fluorescent beads. During analysis, the absolute number (cells/µL) of positive cells in the sample can be determined by comparing cellular events to bead events. Figure 3 summarises the phenotypic characterisation of T lymphocyte subsets and the antibodies used in the FACS analysis.^23^

### Outcome measures and analysis

#### Image analysis

The table above lists the endpoints for this study (table 2). The primary endpoint in this study will be the change in vascular inflammation as measured by mean TBR_max_ in the index vessel on ^18^F-FDG PET/CT scans from baseline to follow-up. This will be calculated by demarcating a region of interest (ROI) including arterial wall and lumen on each axial slice of artery (ascending aorta and both carotid arteries) on the coregistered PET/CT scan and maximum standardised uptake values recorded. Subsequently, ROIs will be normalised by the blood pool ^18^F-FDG concentration in the superior vena cava or jugular vein (for carotids) and averaged to yield an arterial mean TBR_max_ as a quantitative measure of arterial tracer uptake. The ‘index vessel’ defined as the arterial territory with the highest mean TBR_max_ at baseline will be the primary outcome variable.^40^ The change in mean TBR_max_ will be calculated for each index vessel from baseline to follow-up and compared between the treatment groups.

**Table 2:** IVORY study endpoint

Primary endpoints	Change in vascular inflammation (as measured by mean TBR_max_ in the index vessel) on ^18^F-FDG PET/CT from baseline to follow-up scans.
Secondary endpoints	Change in mean TBR_max_ in each arterial region individually restricted to those slices with TBR >1.6. Change in lymphocyte subsets between low-dose IL-2 and placebo. Change in percentage of Treg cells between low-dose IL-2 and placebo. The safety and tolerability of extended dosing of IL-2 in patients with ACS will be evaluated.
Exploratory endpoints	The change in serum cardiac biomarkers (eg, hsCRP, troponin, interleukin 6). Change in ejection fraction as measured on transthoracic echocardiograms. Change in phenotype and function of peripheral blood mononuclear cell subsets (such as B lymphocytes and natural killer cells) as assessed by flow cytometry, gene expression and in vitro activation and suppression assays.

ACS, acute coronary syndrome; ^18^F-FDG, 2-deoxy-2-[fluorine-18] fluoro-D-glucose; hsCRP, high-sensitivity C reactive protein; IL-2, interleukin 2; PET, positron emission tomography; TBR_max_, maximum target to background ratio; Treg cells, regulatory T cells.

**Figure 4:**
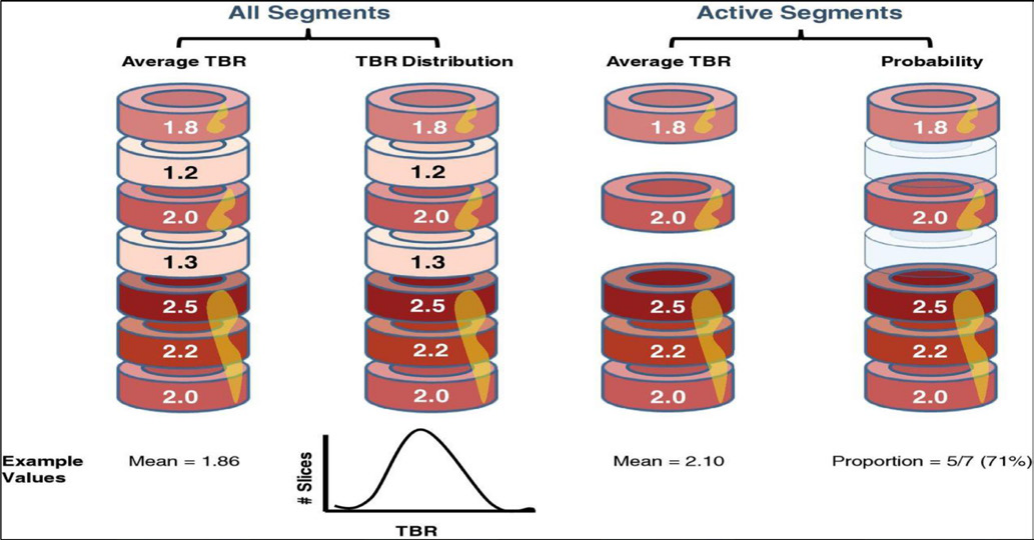
Analysis methods used to quantify vascular inflammation in the index vessel. TBR, target to background ratio.

Furthermore, an analysis of change from baseline in average TBR_max_ for active segments within the index vessel will be carried out, as previously described (figure 4).^35^ An active segment will be defined as a segment of a vessel with a TBR of ≥1.6. An analysis of the probability of a segment being active within the index vessel will also be calculated.

Change from baseline in average TBR_max_ will be analysed using analysis of covariance, fitting treatment as a fixed effect and including baseline value and ST-elevation status as covariates. Point estimates and corresponding 95% CIs will be constructed with p-values for the relevant comparisons of interest. All nominal p-values will be reported. Multiple testing across both analyses will be controlled using a gate-keeping approach. Multiple testing corrections will be carried out to obtain the p-values.

Average mean TBR_max_ data from the final imaging visit will be plotted to show the distribution from all segments from all index vessels within each treatment group. The difference between low-dose IL-2 and placebo will be calculated and tested using a non-parametric permutation test at an individual patient level.

The number of active segments and the total number of segments will be included in logistic regression analyses to model the probability of a segment being active. For baseline correction within each group, a model will be fitted with terms for treatment and visit. For placebo and baseline correction, a model will be fitted with treatment term, including the baseline proportion of active segments as a covariate.

#### Lymphocyte subset and biomarker analysis

A detailed lymphocyte subset analysis will be performed to determine the change in percentage of Treg cells (CD3^+^ CD4^+^ CD8^-^ CD25^high^ CD127^low^) between low-dose IL-2 and placebo throughout the treatment period using flow cytometry. Other lymphocyte subsets will also be assessed in a similar manner.

The change in serum cardiac biomarkers will be evaluated by blood sample analysis at various time points in the trial. These will include high-sensitivity troponin I (hs-Trop I), hsCRP and IL-6. hsCRP at screening and all hs-Trop I measurements will be analysed by the Department of Biochemistry/Clinical Immunology at CUH. All other biomarkers will be analysed by the Core Biochemical Assay laboratory, Cambridge, UK.

Categorical variables will be reported as percentages and counts. All continuous variables will be reported as mean, median, SD, minimum and maximum values. The full analysis population will include all patients who received the trial drug or placebo and completed the treatment course after randomisation.

### Adverse event (AE) reporting

Safety and tolerability of low-dose IL-2 in the ACS population will be monitored throughout the course of the study. All AEs will be documented, and a medical doctor blinded to the treatment allocation will determine the causality of each in conjunction with the trial Principal Investigator. AEs, adverse reactions (ARs), serious AEs/serious ARs (SAEs/SARs) and suspected unexpected SARs will be defined as per the International Conference on Harmonisation definitions. SAEs, SARs, suspected unexpected serious adverse reactions will be subject to expedited reporting as per the Medicines and Healthcare products Regulatory Agency guidelines. Severe ARs and SARs will also be documented on a separate log, as these are part of the trial stopping criteria. For all AEs, an assessment of severity, date of onset and resolution will be also documented. Abnormal laboratory results will only be documented as AEs if they are deemed to be of clinical significance.

An independent data monitoring committee (iDMC) will be responsible for the review of all unblinded safety data and will meet quarterly for the first year of the trial and every 6 months until the follow-up visit of the last patient. All iDMC meetings will be held without the involvement of the trial team apart from the unblinded statistician. The trial management group will also assess safety in a blinded manner on an ongoing basis during the trial.

#### Trial stopping criteria

For the first 10 patients dosed, if a cumulative total of three SARs or severe ARs are observed the trial will be halted. This will trigger an unscheduled iDMC meeting to review the unblinded trial data to date.

After the 10th patient has commenced dosing, a percentage basis (30%) of patients who experience prespecified events (SARs or severe ARs) will be used to halt the trial and trigger a review by the iDMC. Once the trial has halted, it will only resume after the approval of a substantial amendment to the regulatory authorities that have approved the trial.

### Patient and public involvement

Patient and public representatives were involved in the design of this study protocol. The NIHR Cambridge BRC Communications and PPI/E Department for Patient and Public Involvement reviewed the patient facing documents and their feedback has been incorporated as much as possible.

## Ethics and dissemination

The Health Research Authority and Health and Care Research Wales, UK (19/YH/0171), approved the study. All participants are required to read the patient information sheet in full and give written consent to participate in the trial. Annual reports will be submitted to the research ethics committee in accordance with national requirements. The results will be reported through peer-reviewed journals and conference presentations.

## Supplementary Material

Reviewer comments

Author's manuscript
