# Supplementary material for: Low-dose interleukin 2 for the reduction of vascular inflammation in acute coronary syndromes (IVORY): protocol and study rationale for a randomised, double-blind, placebo-controlled, phase II clinical trial
Source: BMJ Open. 2022 Oct 7;12(10):e062602. doi: 10.1136/bmjopen-2022-062602 (PMC9558794; doi:10.1136/bmjopen-2022-062602)
Supplement: Supplementary data [file bmjopen-2022-062602supp002.pdf]

Patient Informed Consent form

Trial Title: Low-dose interleukin-2 for the reduction of vascular inflammation in Acute Coronary Syndromes (IVORY)

Principal Investigator: **Participant Number: __________**

If you agree with each sentence below, please initial the box **INITIALS**

1	I have read and understood the Participant Information Sheet version 3.0, dated 01 March 2021 for the above trial and I confirm that the trial procedures and information have been explained to me. I have had the opportunity to ask questions and I am satisfied with the answers and explanations provided.	
2	I understand that my participation in this trial is voluntary and that I am free to withdraw at any time, without giving a reason and without my medical care or legal rights being affected.	
3	I understand that personal information about me will be collected and used in accordance with this information sheet. This information will be kept in the strictest confidence and none of my personal data will be published.	
4	I understand that sections of my medical notes or information related directly to my participation in this trial may be looked at by responsible individuals from the sponsor, regulatory authorities and research personnel where it is relevant to my taking part in research and that they will keep my personal information confidential. I give permission for these individuals to have access to my records.	
5	I understand that my GP will be informed of my participation and any incidental clinical findings of relevance in this trial as well as information about the IVORY trial.	
6	I understand that my personal data might be transferred between the trial team at different trial sites in relation to my participation in this trial. I understand that any personal data will be sent using (secure/encrypted mail servers etc).	
7	I have read and understood the compensation arrangements for this trial as specified in the Participant Information Sheet.	
8	I understand that the doctors in charge of this trial may close the trial, or stop my participation in it at any time without my consent.	
9	I have read and understood my responsibilities for the trial including using appropriate contraception as listed in section 6.	
10	I understand that any data and samples already collected will still be used by the trial team if I withdraw/am withdrawn from the trial, unless I instruct the team to destroy them.	

OPTIONAL **YES NO**

		YES	NO
11	I agree that in the event that there are any remaining unused samples at the end of the IVORY trial analysis these may be used in other approved research studies.		
12	I give my permission to be re-contacted in the future by this research team and other research teams to invite me to take part in new studies that may be of interest. I understand that this does not oblige me to take part in further research.		

I agree to participate in this trial:

______________________________	_____________________________________	______________
Name of patient	Signature	Date
______________________________	_____________________________________	______________
Name of person taking consent	Signature	Date

Time of Consent (24hr clock) _______:_______

1 copy for the patient, 1 copy for the trial team, 1 copy to be retained in the hospital notes.
