# Supplementary material for: Low-dose interleukin 2 for the reduction of vascular inflammation in acute coronary syndromes (IVORY): protocol and study rationale for a randomised, double-blind, placebo-controlled, phase II clinical trial
Source: BMJ Open. 2022 Oct 7;12(10):e062602. doi: 10.1136/bmjopen-2022-062602 (PMC9558794; doi:10.1136/bmjopen-2022-062602)
Supplement: Supplementary data [file bmjopen-2022-062602supp001.pdf]

Supplementary Material

Supplementary Table 1 :
IVORY Inclusion and Exclusion Criteria

**Inclusion Criteria**	• Able to provide written informed consent to participate<br>• Aged between 18 and 85<br>• Current admission (on the screening visit) with an acute coronary syndrome - ST elevation myocardial infarction (STEMI), non-ST elevation myocardial infarction (NSTEMI), or unstable angina (UA) with symptoms suggestive of myocardial ischaemia lasting 10 minutes or longer with the patient at rest or with minimal effort<br>AND EITHER<br>i. elevated levels of troponin on admission<br>OR<br>ii. dynamic changes in ECG (new ST-T changes or T-wave inversion).<br><br>• Where applicable, to be included in the trial women must be<br>i) Postmenopausal (for the purposes of this trial, postmenopausal is defined as being amenorrhoeic for greater than 2 years with an appropriate clinical profile, e.g., age appropriate, history of vasomotor symptoms)<br>1. OR<br>ii) Have had a documented hysterectomy and/or bilateral oophorectomy or sterilised<br>1. OR<br>iii) Perimenopausal with a negative pregnancy test at screening (for the purposes of inclusion in this trial, perimenopausal is defined as women with an appropriate clinical profile, e.g., age appropriate, history of vasomotor symptoms, irregular periods). They will also have to comply with the use of contraception for the duration of the trial and undergo additional pregnancy tests during the trial.<br><br>• High sensitivity C-reactive protein of >2 mg/L at screening<br>• Willingness and possibility to start dosing within 14 days from initial date of admission to the primary hospital for ACS<br>• Able to comply with all trial mandated visits.
**Exclusion Criteria**	• Current presentation (at screening) with cardiogenic shock (systolic blood pressure <80 mmHg, unresponsive to fluids, or necessitating catecholamines).<br>• Current presentation with cardiac arrest<br>• Signs or symptoms of active infection requiring intravenous antibiotic treatment at screening<br>• History of malignancies requiring active treatment (however, patients with a history of treated localised basal or squamous cell skin cancer are not excluded from participation in this trial)<br>• History of solid organ transplantation or other bone marrow transplantation<br>• History of recurrent epileptic seizures in the previous 4 years; repetitive or difficult to control seizures, coma or toxic psychosis lasting >48 hours<br>• Uncontrolled hypotension (systolic blood pressure <80mmHg or diastolic blood pressure <50mmHg) OR uncontrolled hypertension (systolic blood pressure>180mmHg or diastolic blood pressure>120mmHg) at screening<br>• Average corrected QT interval (QTc) > 450msecs using Bazett's formula from average of triplicate ECGs or >480msecs if bundle branch block is present.<br>• Renal impairment defined as creatinine clearance [Cockcroft-Gault] <45ml/min at screening<br>• Liver dysfunction. Defined as alanine transaminase (ALT) >2 x upper limit of normal (ULN) at screening<br>• Evidence of cholestasis defined as elevated total bilirubin levels (TBL) > 1.5 x ULN and alkaline phosphatase (ALP) >1.5 x ULN at screening.<br>• Known hypothyroidism or hyperthyroidism<br>• Known autoimmune disease requiring active immunosuppressive treatment<br>• Any oral or intravenous immunosuppressive treatment including regular prednisolone, hydrocortisone or disease modifying drugs. [Inhaled or topical steroids are permissible]<br>• Patients on cytotoxic drugs and interferon-alpha<br>• Diabetics on oral hypoglycaemics/diet control with a haemoglobin A1c (HbA1c) diabetes control and complications assay (DCCT) > 8% OR HbA1c standardised to

international federation of clinical chemistry and laboratory medicine (IFCC) >64mmol/mol, at screening. Diabetics on insulin are excluded from the study.

- Contraindication to IL-2 treatment or hypersensitivity to IL-2 or to any of its excipients
- Participation in a previous research trial in the last 3 years which involved exposure to significant ionising radiation (i.e., cumulative research radiation dose >5mSv)
- Participation in a clinical trial where the patient has received a drug or new chemical entity within 30 days or 5 half-lives, or twice the duration of the biological effect of the drug (whichever is longer) prior to the first dose of trial medication.
- Any medical history or clinically relevant abnormality that is deemed by the principal investigator (PI)/delegate to make the patient ineligible for inclusion because of a safety concern
- Pregnant women or breast-feeding women
- Patients who are COVID-19 PCR positive at the time of screening
- Known severe allergy to the CT-contrast agents.
