# Supplementary material for: Low-dose interleukin 2 for the reduction of vascular inflammation in acute coronary syndromes (IVORY): protocol and study rationale for a randomised, double-blind, placebo-controlled, phase II clinical trial
Source: BMJ Open. 2022 Oct 7;12(10):e062602. doi: 10.1136/bmjopen-2022-062602 (PMC9558794; doi:10.1136/bmjopen-2022-062602)
Supplement: Author's manuscript [file bmjopen-2022-062602.draft_revisions.pdf]

BMJ Open is committed to open peer review. As part of this commitment we make the peer review history of every article we publish publicly available.

When an article is published we post the peer reviewers' comments and the authors' responses online. We also post the versions of the paper that were used during peer review. These are the versions that the peer review comments apply to.

The versions of the paper that follow are the versions that were submitted during the peer review process. They are not the versions of record or the final published versions. They should not be cited or distributed as the published version of this manuscript.

BMJ Open is an open access journal and the full, final, typeset and author-corrected version of record of the manuscript is available on our site with no access controls, subscription charges or pay-per-view fees (http://bmjopen.bmj.com).

If you have any questions on BMJ Open's open peer review process please email info.bmjopen@bmj.com

BMJ Open

Low-dose interleukin-2 for the reduction of vascular inflammation in acute coronary syndromes (IVORY): protocol and study rationale for a randomised, double-blind, placebo-controlled, Phase II clinical trial.

Journal:	*BMJ Open*
Manuscript ID	bmjopen-2022-062602
Article Type:	Protocol
Date Submitted by the Author:	03-Apr-2022

Complete List of Authors:	Sriranjan, Rouchelle; University of Cambridge, Department of Medicine, Division of cardiovascular medicine<br>Zhao, Tian; University of Cambridge Medicine, Division of Cardiovascular Medicine<br>Tarkin, Jason; University of Cambridge, Department of Medicine, Division of Cardiovascular Medicine<br>Hubsch, Annette; University of Cambridge, Department of Medicine, Division of Experimental Medicine and Immunotherapeutics (EMIT)<br>Helmy, Joanna; University of Cambridge Medicine, Experimental Medicine and Immunotherapeutics (EMIT) Division<br>Vamvaka, Evangelia; University of Cambridge, Department of Medicine, Division of Experimental Medicine and Immunotherapeutics (EMIT)<br>Jalaludeen, Navazh; University of Cambridge, Department of Medicine, Division of Experimental Medicine and Immunotherapeutics (EMIT)<br>Bond, Simon; Cambridge University Hospitals NHS Foundation Trust, Cambridge Clinical Trials Unit<br>Hoole, Stephen; Papworth Hospital NHS Foundation Trust, Cardiology<br>Knott, Philip; Cambridge University Hospitals NHS Foundation Trust, Department of Clinical Immunology<br>Buckenham, Samantha; Cambridge University Hospitals NHS Foundation Trust, Department of Immunology<br>Warnes, Victoria; Cambridge University Hospitals NHS Foundation Trust, Department of Nuclear Medicine<br>Bird, Nick; Cambridge University Hospitals NHS Foundation Trust<br>Cheow, Heok; Cambridge University Hospitals NHS Foundation Trust, Department of Nuclear Medicine<br>Templin, Heike; Cambridge University Hospitals NHS Foundation Trust, Cambridge Clinical Trials Unit<br>Cacciottolo, Paul; University of Cambridge, Department of Medicine,Division of Experimental Medicine and Immunotherapeutics (EMIT)<br>Rudd, James; University of Cambridge Medicine, Division of Cardiovascular Medicine<br>Mallat, Ziad; University of Cambridge Medicine, Division of Cardiovascular Medicine<br>Cheriyan, Joseph; University of Cambridge Medicine, Division of Experimental Medicine & Immunotherapeutics

Keywords:	Cardiology < INTERNAL MEDICINE, Cardiovascular imaging < RADIOLOGY & IMAGING, Immunology < NATURAL SCIENCE DISCIPLINES, Clinical trials < THERAPEUTICS

SCHOLARONE™
Manuscripts

Low-dose interleukin-2 for the reduction of vascular inflammation in acute coronary syndromes (IVORY): protocol and study rationale for a randomised, double-blind, placebo-controlled, Phase II clinical trial.

Rouchelle Sriranjan[1], Tian Zhao[1], Jason Tarkin[1], Annette Hubsch[2], Joanna Helmy[2], Evangelia Vamvanka[2], Navazh Jalaludeen[2], Simon Bond[3], Stephen Hoole[4], Philip Knott[5], Samantha Buckenham[5], Victoria Warnes[6], Nick Bird[6], Heok Cheow[6], Heike Templin[3], Paul Cacciottolo[2], James H.F. Rudd[1]*, Ziad Mallat[1]*, Joseph Cheriyan[2,3]*

***Joint Senior Authors**

CORRESPONDENCE TO:

Dr. Rouchelle Sriranjan: rfss2@cam.ac.uk
Department of Medicine
Division of Cardiovascular Medicine
University of Cambridge Medicine, Cambridge, UK
Addenbrooke's Hospital
Hills Road
CAMBRIDGE
CB2 2QQ

Dr Joseph Cheriyan: jc403@medschl.cam.ac.uk
The Experimental Medicine & Immunotheraputics Unit
Level 3, Addenbrooke's Centre for Clinical Investigation (ACCI)
Box 110
Addenbrooke's Hospital
Hills Road
CAMBRIDGE
CB2 2QQ

AUTHOR AFFILIATIONS

[1]Department of Medicine, Division of Cardiovascular Medicine, University of Cambridge, Cambridge, UK
[2]Division of Experimental Medicine and Immunotherapeutics (EMIT), Department of Medicine, University of Cambridge, Cambridge, UK
[3]Cambridge Clinical Trials Unit, Cambridge University Hospitals, Cambridge, UK
[4]Department of Interventional Cardiology, Royal Papworth Hospital NHS Trust, Cambridge, UK
[5]Department of Immunology, Cambridge University Hospitals, Cambridge, UK
[6]Department of Nuclear Medicine, Cambridge University Hospitals, Cambridge, UK

Word count: 3887

For peer review only

ABSTRACT

Introduction: Inflammation plays a critical role in the pathogenesis of atherosclerosis, the leading cause of ischemic heart disease (IHD). Studies in pre-clinical models have demonstrated that an increase in regulatory T cells (Tregs), which have a potent immune modulatory action, led to a regression of atherosclerosis. The Low-dose InterLeukin-2 (IL-2) in patients with stable ischaemic heart disease and Acute Coronary Syndromes (LILACS) study, established the safety of low-dose IL-2 and its biological efficacy in IHD. The IVORY trial is designed to assess the effects of low-dose IL-2 on vascular inflammation in patients with acute coronary syndromes (ACS).

Methods and analysis: In this study, we hypothesize that low-dose IL-2 will reduce vascular inflammation in patients presenting with ACS. This is a double-blind, randomised, placebo-controlled, phase II clinical trial. Sixty ACS (unstable angina, non-ST elevation, or ST elevation myocardial infarction) patients with high sensitivity CRP (hsCRP) levels >2mg/L, will be randomised to receive either 1.5 x 10^6IU of low-dose IL-2 or placebo (1:1). Dosing will commence within 14 days of admission. Dosing will comprise of an induction and a maintenance phase, spanning across 8 weeks. 2-deoxy-2-[fluorine-18] fluoro-D-glucose (^{18}F-FDG) positron emission tomography/ computed tomography (PET/CT) scans will be performed before and after dosing. The primary endpoint is the change in mean maximum target to background ratios (TBR_{max}) in the index vessel between baseline and follow-up scans. Changes in circulating T cell subsets will be measured as secondary endpoints of the study. The safety and tolerability of extended dosing with low-dose IL-2 in patients with ACS will be evaluated throughout the study.

Ethics and dissemination: The health research authority and health care research wales, UK (19/YH/0171), approved the study. The results will be reported through peer-reviewed journals and conference presentations.

Trial registration number EudraCT 2017-005130-27; Protocol date: 01.03.2021, version 3.0, Approval 08.03.2021.

For peer review only

Strengths and Limitations of this study

- **This is the first study to explore the clinical efficacy of low-dose IL-2 in reducing vascular inflammation in patients presenting with ACS.**
- **This is the first study to explore the safety of extended dosing with low-dose IL-2 in patients presenting with ACS.**
- **Changes in circulating lymphocyte subsets with extended treatment with low-dose IL-2 have not been studied in this population before.**
- **This is the first cardiovascular interventional study to be done using ^{18}F-FDG PET/CT in acutely presenting ACS patients.**
- **Due to the early phase nature of this study, it is not powered to determine if treatment with low dose IL-2 would lead to improvements in clinical outcomes.**

INTRODUCTION

ATHEROSCLEROSIS AND INFLAMMATION

Inflammation plays a pivotal role in the initiation and progression of atherosclerosis, which accounts for an overwhelming majority of cardiovascular events. The role of the immune system in the pathophysiology of atherosclerosis and plaque instability is multi-faceted and orchestrated by both, the innate and adaptive components of the immune system.[1] The Canakinumab ANti-inflammatory Thrombosis Outcomes Study (CANTOS) reported for the first time that anti-inflammatory therapy significantly reduced recurrent cardiovascular events in patients with IHD and ongoing inflammation with persistently raised hsCRP levels.[2] Further efforts to harness the immune system for pharmacological therapeutic strategies in cardiovascular disease (CVD) are currently underway.[3 4]

Due to their critical role in mediating immune tolerance and anti-atherosclerotic effects, natural and inducible Treg cells ($CD4^{+}CD25^{+}$FOPX3) are a focus of novel therapeutic strategies. Initial studies in pre-clinical models showed that Tregs reduced plaque inflammation, slowed progression and even led to regression of atherosclerosis.[5 6] Tregs have also been implicated in moderating the post-ischaemic immune responses triggered by self-antigens presented by necrotic myocardium.[7] In line with this finding, it has also been demonstrated that Tregs promote myocardial wound healing, attenuate adverse left ventricular remodelling and improve cardiac function. [8-11] The Malmo Diet and Cancer study further demonstrated an increased risk of acute coronary events in patients with low levels of baseline circulating Treg cells.[12] Observational studies have also shown that patients with ACS have lower numbers of circulating Tregs with impaired function.[13-16] Furthermore, low levels of Tregs maybe also be associated with plaque instability.[15]

Interleukin-2 (IL-2) plays a crucial role in immune homeostasis by determining the balance between T effector (Teffs) and Tregs.[17 18] Whilst IL-2 is implicated in the development of both Teffs and Tregs, unlike Teffs, Treg cells constitutively express an IL-2 receptor (IL-2Rα) which binds to IL-2 with high affinity, making this subset

very sensitive to IL-2.[19] Of the cytokines involved in Treg development and stability, IL-2 is the most essential[20].

The administration of low dose IL-2 has been used in autoimmune conditions such as graft versus host disease (GVHD), autoimmune vasculitis, hepatitis, type 1 diabetes mellitus (T1DM), and systemic lupus erythematosus (SLE), to boost Treg numbers with promising results.[17 21-24] Furthermore, a recent study involving patients with 11 auto-immune conditions demonstrated the clinical efficacy and safety of low-dose IL-2.[25]

LILACS

The Low dose InterLeukin-2 in patients with stable ischaemic heart disease and Acute Coronary Syndromes (LILACS) was the first study to explore the safety of low dose of IL-2 and its ability to expand Treg cells in this patient cohort. This was a double blinded, randomised controlled adaptive Phase I/IIa trial.[26] Twenty-five patients with stable IHD were dosed in part A with aldesleukin (recombinant IL-2). The dose range used in this trial was between 0.3-3 x 10^6IU. The ratio of IL-2 to placebo was 3:2 for each group. An increase in Treg cells was observed at the doses tested without any significant increases in Teff cells.[26] Part B, which included patients with ACS (non-ST elevation myocardial infarction and unstable angina), was carried out upon completion of part A. Two doses (1.5 and 2.5 x 10^6IU) were used in part B (IL-2: placebo = 6:2). In both parts, the low-dose IL-2 and placebo were administered for 5 consecutive days. Low-dose IL-2 was well tolerated in both stable IHD and ACS patients. The most commonly occurring adverse reactions were injection site reactions, which were temporary. Other adverse reactions included flu like symptoms and myalgia. No serious adverse events (SAEs) were observed in part A. Two SAEs were noted in part B that were deemed to be unrelated to the investigational medical product (IMP). In LILACS, the target dose needed to achieve a 75% increase in Tregs was determined as 1.46 x10^6IU.

Based on the promising results of LILACS, we hypothesized that low-dose IL-2 would reduce vascular inflammation in patients with ACS as assessed by ^{18}F-FDG PET/CT imaging before and after treatment.

^{18}F-FDG is the most widely used radionuclide in imaging inflammation in the context of atherosclerosis. Limitations of ^{18}F-FDG include the fact that it cannot be used to quantify inflammation in coronary arteries reliably and that the ^{18}F-FDG signal is not inflammatory cell specific as all glucose metabolising cells take up ^{18}F-FDG.[27-29] However, with respect to atherosclerosis, *ex vivo* histological studies from arterial specimens have shown a strong correlation between ^{18}F-FDG uptake and macrophage density.[30 31] This is thought to be due to increased glucose turnover in macrophages compared to other cells within the atherosclerotic plaque. Furthermore ^{18}F-FDG quantification is highly reliable and reproducible in large to medium sized vessels.[32] ^{18}F-FDG PET/CT has been used by our group and others as a biomarker for drug development in Phase II cardiovascular drug intervention studies.[33-35] ^{18}F-FDG PET/CT has specifically been used to study the effect of statins and anti-inflammatory compounds on vascular inflammation.[36-40]

METHODS

The IVORY trial is a prospective, randomised, double-blind, placebo-controlled, Phase II clinical trial. This is an academic study involving two centres namely, Cambridge University Hospitals (CUH) and Royal Papworth Hospital (RPH), in Cambridge, UK. Overall study coordination, monitoring and auditing will be undertaken by the Cardiovascular Trials Office of the Cambridge Clinical Trials Unit (CCTU), CUH. The study is sponsored by CUH.

STUDY POPULATION

To be included, participants must be admitted with a diagnosis of ACS. The study will include patients presenting with ST elevation myocardial infarction (STEMI), non-ST elevation myocardial infarction (NSTEMI), or unstable angina (UA). The participants should have symptoms suggestive of myocardial ischaemia lasting 10 minutes or longer at rest or on minimal effort; and either have elevated levels of troponin on admission and/or dynamic electrocardiogram (ECG) changes suggestive of ischaemia. Furthermore, to be included participants will require hsCRP levels >2mg/L at screening. We hypothesize patients presenting with hsCRP levels >2mg/L have residual systemic inflammation and are at higher risk of recurrent cardiovascular events. Therefore, recruitment will be restricted to these patients as they are most likely to benefit from this treatment.

Due to the ionising radiation associated with ^{18}F-FDG PET/CT scans, to be included in the trial, women must be either postmenopausal, have had a documented hysterectomy and/or bilateral oophorectomy or sterilisation procedure, or be perimenopausal with a negative pregnancy test at screening. Perimenopausal women will also have to comply with the use of contraception for the duration of the trial and undergo additional pregnancy tests during and after treatment.

Exclusion criteria include patients who present with cardiogenic shock, cardiac arrest, and uncontrolled hypertension/hypotension. Blood tests will be carried out to exclude patients with thyroid, severe renal, and hepatic dysfunction as specified in the protocol. Patients with active infection, history of malignancy requiring active treatment or on oral/intravenous immune suppression therapy will also be excluded. Patients with type 1, or type 2 diabetes mellitus (T2DM) with poor control, will be excluded due to the challenges in analysing ^{18}F-FDG PET/CT scans in this patient cohort.[40] Due to the ongoing pandemic and to minimise potential risk, patients who are coronavirus disease 2019 (COVID-19) positive on polymerase chain reaction (PCR) at the time of screening will not be recruited to the trial. Detailed inclusion and exclusion criteria are listed in supplementary table 1.

We plan to recruit sixty patients for the IVORY study. The sample size is based on an absolute difference of 0.2 in the primary endpoint (mean TBR_{max} in the index vessel) between placebo and active treatment at the end of the dosing period. This is equivalent to a 10% difference from a reference value of 2.02 and equivalent to the size effect observed after atheroprotective therapy.[35-37] This is based on the results of previous studies, that have reported less than a 10% difference in mean TBR_{max} in the index vessel between active treatment and placebo and failed to reduce CV outcomes[34 39 41-43].

For peer review only - http://bmjopen.bmj.com/site/about/guidelines.xhtml

Assuming a SD of 0.24, 24 patients per arm, testing at 2-sided 5% significance level, would provide 80% power. Therefore, a sample size of 30 completed patients per arm was selected to account for participant dropout and poor quality scans deemed to be unsuitable for analysis (e.g., because of motion artifact).

DOSE SELECTION FOR IVORY

A dose of 1.5 x 10^6IU was selected for the study based on analysis of data from the LILACS trial. In ACS patients dosed in Part B of the LILACS trial, a dose of 1.5 x 10^6IU led to an 80% increase in Tregs after five consecutive days of treatment. No significant increases in Teff cells were noted at this dose.

Active drug description
Aldesleukin is produced by recombinant DNA technology using an *Escherichia coli* strain that contains a genetically engineered modification of the human IL-2 gene. Commercially available aldesleukin with an UK marketing authorisation will be used and initially prepared as per the manufacturer's guidance. This preparation will be diluted further using 5% glucose, to give a final concentration of 3.3 x 10^6 IU/ml. The administration of aldesleukin will be by subcutaneous (S/C) injection.

Placebo description
Commercially available dextrose 5% with an UK marketing authorisation at equivalent dose volume will be used for the placebo formulation.

STUDY PROTOCOL

The trial will be performed in accordance with the spirit and the letter of the declaration of Helsinki, the conditions and principles of Good Clinical Practice, the protocol and applicable local regulatory requirements and law. Participants will be provided with an information sheet and given the opportunity to consider the information for at least for 24 hours. They will have an opportunity during this time to discuss the study with trial staff and have any queries answered before consenting to participate in the trial. Full informed consent (Supplementary file 1) will be taken at the screening visit (V1) by the study team. The screening visit will also include baseline blood tests, a physical examination, vitals (blood pressure, heart rate, temperature), a clinical history, and ECGs in triplicate. The screening bloods will include safety blood tests (full blood count; electrolytes/urea/creatinine; liver function tests), thyroid function tests, hsCRP, serum/urine pregnancy test where applicable and a full lipid profile.

Each participant who passes screening will be assigned an ID number. The research team will complete a case record form (CRF) for each encounter with the participant. The data is then sent to the CCTU for entry into the electronic trial database. All CRFs will be stored safely, and patient identifiable information is kept confidential.

Randomisation will then be carried out using sealed envelope (www.sealedenvelope.com). Randomisation will be stratified by ST elevation status to minimise bias between the groups since ST-elevation MI patients typically have higher circulating hsCRP levels.

For peer review only

During the trial, the patient facing trial team, patients and data analysis teams (immunology sample analysers and image analysers) are blinded to the treatment allocation. To maintain the overall quality and legitimacy of the clinical trial, unblinding will only occur in exceptional circumstances when knowledge of the actual treatment is essential for further clinical management of the patient.

Figure 1: Summary of Trial Design

The trial design is summarised in Figure 1. After randomisation, patients will undergo an ^{18}F-FDG PET/CT scan covering the ascending aorta and carotid arteries (V2). The ^{18}F-FDG PET/CT scans will be carried out in CUH. Patients would be instructed to fast for 6 hours prior to scanning. A finger prick blood sugar test will be performed prior to the PET/CT scan. A carotid angiogram will be undertaken after the PET/CT scan where possible. The carotid angiogram can be performed either in visit 2 or 15, as this is for anatomical correlation to enable more accurate measurement of ^{18}F-FDG uptake in the carotid arteries. A transthoracic echocardiogram will also be undertaken prior to dosing to assess the left ventricular function and regional wall motion abnormalities.

Dosing will commence within 14 days of admission, and after the baseline ^{18}F-FDG PET/CT scan (Figure 2). Dosing will consist of two phases spanning a period of 8 weeks. The induction phase will involve once daily S/C injections of IMP for 5 days (V3-V7). During the maintenance phase participants will receive once weekly S/C injections of IMP for 7 weeks. Dosing visits will last up to 1 hour during the induction phase. At each visit, participants will be screened for potential side-effects and their medications will be reviewed by a clinician. Participants will also undergo a physical examination, have their vital signs measured, have ECGs recorded, and safety bloods taken. Circulating systemic inflammatory biomarkers (hsCRP, interleukin-6), cardiac troponin I and lymphocyte subsets will be measured on V3 and V7.

Figure 2: IVORY trial visits – timeline and assessments

During the maintenance phase (V8-V14), participants will be dosed once weekly. These visits will last 1-1.5 hours. They will be similar to the induction phase visits; however, administration of the IMP will only occur once safety bloods taken during the visit are checked. Cardiac biomarkers and lymphocyte subsets will be measured during alternate maintenance visits (V8, V10, V12, V14). Within approximately 1 week from the completion of dosing, a second post-dose ^{18}F-FDG PET/CT scan will be undertaken. The final encounter will take place approximately 4 weeks after the final dosing visit via telephone unless an in-person visit is necessary for clinical or safety reasons.

Patients will be withdrawn from the trial after recruitment if any of the pre-specified general, cardiac, renal, or liver withdrawal criteria are met. They will also be withdrawn from the trial if they are positive for COVID-19 during dosing. The withdrawal criteria are summarised in Table 1.

Table 1: IVORY study withdrawal criteria

For peer review only - http://bmjopen.bmj.com/site/about/guidelines.xhtml

**General**	• Cardiorespiratory arrest<br>• Failure to attend 2 scheduled dosing appointments without adequate reason based on the PI assessment<br>• Severe hypersensitivity reactions will preclude any further IMP administration<br>• New seizure activity /Coma<br>• Severe lethargy or somnolence<br>• Respiratory insufficiency requiring intubation<br>• Pregnancy<br>• Withdrawal of consent<br>• PI discretion<br>• Any serious adverse reaction or adverse reaction which is deemed by investigators to be Severe AR.<br>• Any significant incidental finding on PET/CT scan clinical governance reports, which in the opinion of the PI, necessitates further investigation and management.<br>• Any medical history, clinically relevant abnormality or reason that is deemed by the PI to make the patient ineligible to continue the trial.
**Cardiac**	• In patients without left bundle branch block (LBBB) QTc interval >500ms OR change from baseline: QTcB >60ms<br>• In patients with LBBB if baseline corrected QT interval increases from <450ms to >500ms OR from 450-480ms to >530ms.<br>(Withdrawal of patients is to be based on an average QTc value of triplicate ECGs)<br>• Patients who will be scheduled to undergo coronary artery bypass grafting<br>• Patients who develop new onset severe pulmonary oedema/new severe congestive heart failure, requiring high dose > 240 mg over 24 hours IV furosemide during admission<br>• Patients with symptomatic uncontrolled systolic BP <80 mmHg and/or diastolic BP <50 mmHg (after at least 2 repeat recordings), or severe hypertension (as defined by BP > 180 mmHg systolic BP or >120 mmHg diastolic BP on at least two readings)<br>• Patients with sustained (>30sec) or symptomatic ventricular tachycardia and patients with ventricular fibrillation will be withdrawn
**Renal**	• Serum creatinine elevation > 2 x baseline screening visit (V1)
**Liver**	• ALT ≥3x ULN and total bilirubin ≥2xULN<br>• ALT ≥5xULN.<br>• ALT ≥3xULN if associated with symptoms (new or worsening) believed to be related to hepatitis (such as fatigue, nausea, vomiting, right upper quadrant pain or tenderness or jaundice) or hypersensitivity (such as fever, rash, or eosinophilia).<br>• Isolated ALT ≥3xULN that persists for ≥4 weeks

STUDY PROCEDURES

a) ^{18}F-FDG scans

A General Electric Discovery 690 PET/CT scanner (Milwaukee, Wisconsin) or equivalent scanner, will be used for imaging. This will be performed using reproducible, validated methods for image acquisition, reconstruction and interpretation as recommended by the European Association of Nuclear Medicine for the conduct of clinical trials using ^{18}F-FDG PET/CT.[40] A dose of approximately 240Mbq ^{18}F-FDG will be injected through a peripheral venous cannula. The ascending aorta will be imaged 90 minutes after the ^{18}F-FDG injection. Attenuation correction and non-contrast CT scans of the ascending aorta will initially be performed. This will be followed by a single bed PET scan acquired in 3D list mode for 10 minutes, with the

superior portion of the aortic arch as the upper anatomical landmark of the scan. Carotid artery imaging will be undertaken immediately after the PET/CT scans of the ascending aorta. A single bed PET scan will be acquired in 3D list mode for 15 minutes, with the external auditory meatus as the upper anatomical landmark of the scan. PET data for the ascending aorta and carotids will be reconstructed using iterative 3D time of flight ordered-subset expectation maximization with standard corrections applied ± point-spread function modelling to reduce partial volume error. A CT carotid angiogram will also be carried out after the PET acquisition either in visit 2 or visit 15.

All scans will be anonymised. The anonymised scans will be analysed in batches of 20 by the research team. Second person checks will also be performed on a subset of these scans.

b) Lymphocyte subset assays

Lymphocyte subset assays will be performed at the Department of Clinical Immunology, CUH and this will occur within 4 hours of sample collection. The clinical investigators and laboratory technicians will be blinded to treatment allocation.

Whole blood will be assessed by performing fluorescence-activated cell sorting (FACS) to measure the absolute and percentage values for lymphocytes subsets (Teffs, T helper 1, T helper 2, T helper 17 and T follicular helper cells) and Treg cells. 50µl antibody reagent and 50µl whole blood will be added directly to the BD Trucount tube. The lyophilized pellet in the tube dissolves, releasing a known number of fluorescent beads. During analysis, the absolute number (cells/µL) of positive cells in the sample can be determined by comparing cellular events to bead events. Figure 3 summarises the phenotypic characterisation of T lymphocyte subsets and the antibodies used in the FACS analysis.[44]

Figure 3: Phenotypic Characterisation and Antibodies used in fluorescence-activated cell sorting (FACS) analysis of T cell subsets

OUTCOME MEASURES AND ANALYSIS

Image analysis

The table above lists the endpoints for this study (Table 2). The primary endpoint in this study will be the change in vascular inflammation as measured by mean TBR_{max} in the index vessel on ^{18}F-FDG PET/CT scans from baseline to follow up. This will be calculated by demarcating a region of interest (ROI) including arterial wall and lumen on each axial slice of artery (ascending aorta and both carotid arteries) on the co-registered PET/CT scan and maximum standardised uptake values (SUVmax) recorded. Subsequently, ROIs will be normalised by the blood pool ^{18}F-FDG concentration in the superior vena cava or jugular vein (for carotids) and averaged to yield an arterial mean TBR_{max} as a quantitative measure of arterial tracer uptake. The "index vessel" defined as the arterial territory with the highest mean TBR_{max} at baseline will be the primary outcome variable.[40] The change in mean TBR_{max} will be calculated for each index vessel from baseline to follow-up and compared between the treatment groups.

For peer review only

Table 2: IVORY study endpoint

**Primary endpoints**	• Change in vascular inflammation (as measured by mean TBR_{max} in the index vessel) on ^{18}F-FDG PET/CT from baseline to follow up scans
**Secondary endpoints**	• Change in mean TBR_{max} in each arterial region individually restricted to those slices with TBR>1.6<br>• Change in lymphocyte subsets between low dose IL-2 and placebo.<br>• Change in percentage of Treg cells between low dose IL-2 and placebo.<br>• The safety and tolerability of extended dosing of IL-2 in ACS patients will be evaluated
**Exploratory Endpoints**	• The change in serum cardiac biomarkers (e.g., hsCRP, troponin, Interleukin-6).<br>• Change in ejection fraction as measured on transthoracic echocardiograms<br>• Change in phenotype and function of peripheral blood mononuclear cell (PBMC) subsets (such as B lymphocytes and Natural Killer cells) as assessed by flow cytometry, gene expression, and in vitro activation and suppression assays.

Furthermore, an analysis of change from baseline in average TBR_{max} for active segments within the index vessel will be carried out, as previously described (Figure 4).[35] An active segment will be defined as a segment of a vessel with a TBR of ≥1.6. An analysis of the probability of a segment being active within the index vessel will also be calculated.

Figure 4: Analysis methods used to quantify vascular inflammation in the index vessel

Change from baseline in average TBR_{max} will be analysed using analysis of covariance, fitting treatment as a fixed effect, and including baseline value and ST-elevation status as covariates. Point estimates and corresponding 95% confidence intervals (CI) will be constructed with P values for the relevant comparisons of interest. All nominal p-values will be reported. Multiple testing across both analyses will be controlled using a gate-keeping approach. Multiple testing corrections will be carried out to obtain the p-values.

Average mean TBR_{max} data from the final imaging visit will be plotted to show the distribution from all segments from all index vessels within each treatment group. The difference between low-dose IL-2 and placebo will be calculated and tested using a nonparametric permutation test at an individual patient level.

The number of active segments and the total number of segments will be included in logistic regression analyses to model the probability of a segment being active. For baseline correction within each group, a model will be fitted with terms for treatment and visit. For placebo and baseline correction, a model will be fitted with treatment term, including the baseline proportion of active segments as a covariate.

Lymphocyte subset and biomarker analysis

A detailed lymphocyte subset analysis will be performed to determine the change in percentage of Treg cells ($CD3^+$ $CD4^+$ $CD8^-$ $CD25^{high}$ $CD127^{low}$) between low dose IL-2 and placebo throughout the treatment period using flow cytometry. Other lymphocyte subsets will also be assessed in a similar manner.

For peer review only

The change in serum cardiac biomarkers will be evaluated by blood sample analysis at various time points in the trial. These will include high sensitivity troponin I (hs-Trop I), hsCRP and IL-6. HsCRP at screening and all hs-Trop I measurements will be analysed by the Department of Biochemistry/Clinical Immunology at CUH. All other biomarkers will be analysed by the Core Biochemical Assay laboratory, Cambridge.

Categorical variables will be reported as percentages and counts. All continuous variables will be reported as mean, median, SD, minimum and maximum values. The full analysis population will include all patients who received the trial drug or placebo and completed the treatment course after randomisation.

ADVERSE EVENT (AE) REPORTING

Safety and tolerability of low-dose IL-2 in the ACS population will be monitored throughout the course of the study. All AEs will be documented, and a medical doctor blinded to the treatment allocation will determine the causality of each in conjunction with the trial Principal Investigator. AEs, adverse reactions (ARs), serious AE/ARs (SAEs/SARs) and suspected unexpected SARs will be defined as per the International Conference on Harmonisation definitions. SAEs, serious adverse reactions (SARs), suspected unexpected serious adverse reactions (SUSARs) will be subject to expedited reporting as per the Medicines and Healthcare products Regulatory Agency (MHRA) guidelines. Severe ARs and SARs will also be documented on a separate log, as these are part of the trial stopping criteria. For all AEs, an assessment of severity, date of onset and resolution will be also documented. Abnormal laboratory results will only be documented as AEs if they are deemed to be of clinical significance.

An independent data monitoring committee (iDMC) will be responsible for the review of all unblinded safety data and will meet quarterly for the first year of the trial and every six months until the follow-up visit of the last patient. All iDMC meetings will be held without involvement of the trial team apart from the unblinded statistician. The trial management group (TMG) will also assess safety in a blinded manner on an ongoing basis during the trial.

Trial stopping criteria

For the first ten patients dosed, if a cumulative total of three serious adverse reactions (SARs) or severe adverse reactions (ARs) are observed the trial will be halted. This will trigger an unscheduled independent data monitoring committee meeting to review the unblinded trial data to date.

After the tenth patient has commenced dosing, a percentage basis (30%) of patients who experience pre-specified events (SARs or severe adverse reactions) will be used to halt the trial and trigger a review by the iDMC. Once the trial has halted, it will only resume after approval of a substantial amendment to the regulatory authorities that have approved the trial.

PATIENT AND PUBLIC INVOLVEMENT

For peer review only

Patient and public representatives were involved in the design of this study protocol. The NIHR Cambridge BRC Communications and PPI/E Department for PPI reviewed the patient facing documents and their feedback has been incorporated as much as possible.

Annual reports will be submitted to the research ethics committee (REC) in accordance with national requirements.

ACKNOWLEDGEMENTS

RS acknowledges funding from the Medical Research Council UK. TZ acknowledges funding from the British Heart Foundation. JT acknowledges funding from the Wellcome trust (211100/Z/18/Z). ZM acknowledges funding support from the British Heart Foundation (BHF) for his BHF Chair and from Cambridge Biomedical Research Centre. JHFR acknowledges funding support from HEFCE, the NIHR Cambridge Biomedical Research Centre, the British Heart Foundation, the EPSRC and the Wellcome Trust. JC acknowledges funding from The National Institute for Health Research (NIHR) Cambridge Biomedical Research Centre (BRC), which is a partnership between Cambridge University Hospitals NHS Foundation Trust and the University of Cambridge, funded by the National Institute for Health. Research (NIHR). This research was supported by the NIHR Cambridge Biomedical Research Centre (BRC-1215-20014). The views expressed are those of the author(s) and not necessarily those of the NIHR or the Department of Health and Social Care.
The authors acknowledge support from the Cambridge Clinical Research Centre, the Core Biochemistry Assay Laboratory and the Cambridge Clinical Trials Unit in the conduct, analysis and reporting of this clinical trial. The investigators also thank all the patients who participated in this trial, as well as the NHS Trusts and staff who have supported this trial.

FUNDING

This work was funded by the Medical Research Council, grant number MR/N028015/1 and the British Heart Foundation Cambridge Centre of Excellence (RCAG/521). Setup, running of the study and decision to publish results is independent of the funders.

COMPETING INTERESTS None declared.

PATIENT CONSENT Not required.

CONTRIBUTIONS

ZM, JC, JR, RS, TZ, SH, SB, JT, NB, HC were involved in the conception and design of the trial. JC is the principal investigator for this trial. RS,AH, VE, JH, TZ, VW were involved in data collection/acquisition. RS, TZ, SB, JT will be involved in data analysis and finalising the statistical analysis plan. SB and JC will approve the trial Statistical Analysis Plan prior to database lock and analysis

For peer review only

REFERENCES

1. Abdolmaleki F, Hayat SMG, Bianconi V, et al. Atherosclerosis and immunity: a perspective. *Trends in cardiovascular medicine* 2019;29(6):363-71.
2. Ridker PM, Everett BM, Thuren T, et al. Antiinflammatory therapy with canakinumab for atherosclerotic disease. *New England journal of medicine* 2017;377(12):1119-31.
3. Libby P, Everett BM. Novel antiatherosclerotic therapies. *Arteriosclerosis, thrombosis, and vascular biology* 2019;39(4):538-45.
4. Kobiyama K, Saigusa R, Ley K. Vaccination against atherosclerosis. *Current opinion in immunology* 2019;59:15-24.
5. Ait-Oufella H, Salomon BL, Potteaux S, et al. Natural regulatory T cells control the development of atherosclerosis in mice. *Nature medicine* 2006;12(2):178.
6. Sharma M, Schlegel MP, Afonso MS, et al. Regulatory T cells license macrophage pro-resolving functions during atherosclerosis regression. *Circulation research* 2020;127(3):335-53.
7. Xiao J, Yu K, Li M, et al. The IL-2/Anti-IL-2 complex attenuates cardiac ischaemia-reperfusion injury through expansion of regulatory T cells. *Cellular Physiology and Biochemistry* 2017;44(5):1810-27.
8. Hofmann U, Beyersdorf N, Weirather J, et al. Activation of CD4+ T lymphocytes improves wound healing and survival after experimental myocardial infarction in mice. *Circulation* 2012;125(13):1652-63.
9. Matsumoto K, Ogawa M, Suzuki J-i, et al. Regulatory T lymphocytes attenuate myocardial infarction-induced ventricular remodeling in mice. *International heart journal* 2011;52(6):382-87.
10. Dobaczewski M, Xia Y, Bujak M, et al. CCR5 signaling suppresses inflammation and reduces adverse remodeling of the infarcted heart, mediating recruitment of regulatory T cells. *The American journal of pathology* 2010;176(5):2177-87.
11. Yu X, Newland SA, Zhao TX, et al. Innate lymphoid cells promote recovery of ventricular function after myocardial infarction. *Journal of the American College of Cardiology* 2021;78(11):1127-42.
12. Wigren M, Björkbacka H, Andersson L, et al. Low levels of circulating CD4+ FoxP3+ T cells are associated with an increased risk for development of myocardial infarction but not for stroke. *Arteriosclerosis, thrombosis, and vascular biology* 2012;32(8):2000-04.
13. Cheng X, Yu X, Ding Y-j, et al. The Th17/Treg imbalance in patients with acute coronary syndrome. *Clinical immunology* 2008;127(1):89-97.
14. Wolf D, Ley K. Immunity and inflammation in atherosclerosis. *Circulation research* 2019;124(2):315-27.
15. Han S-f, Liu P, Zhang W, et al. The opposite-direction modulation of CD4+ CD25+ Tregs and T helper 1 cells in acute coronary syndromes. *Clinical immunology* 2007;124(1):90-97.
16. Ammirati E, Cianflone D, Banfi M, et al. Circulating CD4+ CD25hiCD127lo regulatory T-Cell levels do not reflect the extent or severity of carotid and coronary atherosclerosis. *Arteriosclerosis, thrombosis, and vascular biology* 2010;30(9):1832-41.

For peer review only

17. Klatzmann D, Abbas AK. The promise of low-dose interleukin-2 therapy for autoimmune and inflammatory diseases. *Nature Reviews Immunology* 2015;15(5):283-94.
18. Zhao TX, Newland SA, Mallat Z. 2019 ATVB plenary lecture: interleukin-2 therapy in cardiovascular disease: the potential to regulate innate and adaptive immunity. *Arteriosclerosis, thrombosis, and vascular biology* 2020;40(4):853-64.
19. Sakaguchi S, Sakaguchi N, Asano M, et al. Immunologic self-tolerance maintained by activated T cells expressing IL-2 receptor alpha-chains (CD25). Breakdown of a single mechanism of self-tolerance causes various autoimmune diseases. *The Journal of Immunology* 1995;155(3):1151-64.
20. Vang KB, Yang J, Mahmud SA, et al. IL-2,-7, and-15, but not thymic stromal lymphopoeitin, redundantly govern CD4+ Foxp3+ regulatory T cell development. *The Journal of Immunology* 2008;181(5):3285-90.
21. Humrich JY, von Spee-Mayer C, Siegert E, et al. Rapid induction of clinical remission by low-dose interleukin-2 in a patient with refractory SLE. *Annals of the rheumatic diseases* 2015;74(4):791-92.
22. Matsuoka K-i, Koreth J, Kim HT, et al. Low-dose interleukin-2 therapy restores regulatory T cell homeostasis in patients with chronic graft-versus-host disease. *Science translational medicine* 2013;5(179):179ra43-79ra43.
23. He J, Zhang X, Wei Y, et al. Low-dose interleukin-2 treatment selectively modulates CD4+ T cell subsets in patients with systemic lupus erythematosus. *Nature medicine* 2016;22(9):991.
24. Koreth J, Matsuoka K-i, Kim HT, et al. Interleukin-2 and regulatory T cells in graft-versus-host disease. *New England Journal of Medicine* 2011;365(22):2055-66.
25. Rosenzwajg M, Lorenzon R, Cacoub P, et al. Immunological and clinical effects of low-dose interleukin-2 across 11 autoimmune diseases in a single, open clinical trial. *Annals of the rheumatic diseases* 2019;78(2):209-17.
26. Zhao T, Sriranjan R, Tuong Z, et al. Regulatory T Cell Response to Low-Dose IL-2 in Ischemic Heart Disease. *NEJM evidence* 2021;1(1)
27. Cheng VY, Slomka PJ, Le Meunier L, et al. Coronary arterial 18F-FDG uptake by fusion of PET and coronary CT angiography at sites of percutaneous stenting for acute myocardial infarction and stable coronary artery disease. *Journal of Nuclear Medicine* 2012;53(4):575-83.
28. Tarkin JM, Joshi FR, Evans NR, et al. Detection of atherosclerotic inflammation by 68Ga-DOTATATE PET compared to [18F] FDG PET imaging. *Journal of the American College of Cardiology* 2017;69(14):1774-91.
29. Folco EJ, Sheikine Y, Rocha VZ, et al. Hypoxia but not inflammation augments glucose uptake in human macrophages: Implications for imaging atherosclerosis with 18fluorine-labeled 2-deoxy-D-glucose positron emission tomography. *Journal of the American College of Cardiology* 2011;58(6):603-14.
30. Ogawa M NS, Saito Y, Kosugi M, Magata Y. What can be seen by 18F-FDG PET in atherosclerosis imaging? The effect of foam cell formation on 18F-FDG uptake to macrophages in vitro. *J Nucl Med* 2012;53(1):55-8.

For peer review only

31. Tawakol A, Migrino RQ, Bashian GG, et al. In vivo 18F-fluorodeoxyglucose positron emission tomography imaging provides a noninvasive measure of carotid plaque inflammation in patients. *J Am Coll Cardiol* 2006;48(9):1818-24.
32. Rudd JH, Myers KS, Bansilal S, et al. Atherosclerosis inflammation imaging with 18F-FDG PET: carotid, iliac, and femoral uptake reproducibility, quantification methods, and recommendations. *Journal of Nuclear Medicine* 2008
33. Tarkin JM, Joshi FR, Rudd JH. PET imaging of inflammation in atherosclerosis. *Nat Rev Cardiol* 2014;11(8):443-57.
34. Fayad ZA, Mani V, Woodward M, et al. Safety and efficacy of dalcetrapib on atherosclerotic disease using novel non-invasive multimodality imaging (dal-PLAQUE): a randomised clinical trial. *Lancet* 2011;378(9802):1547-59.
35. Elkhawad M, Rudd JH, Sarov-Blat L, et al. Effects of p38 mitogen-activated protein kinase inhibition on vascular and systemic inflammation in patients with atherosclerosis. *JACC: Cardiovascular Imaging* 2012;5(9):911-22.
36. Mäki-Petäjä KM, Elkhawad M, Cheriyan J, et al. Anti-tumor necrosis factor-α therapy reduces aortic inflammation and stiffness in patients with rheumatoid arthritis. *Circulation* 2012:CIRCULATIONAHA. 112.120410.
37. Tahara N, Kai H, Ishibashi M, et al. Simvastatin attenuates plaque inflammation: evaluation by fluorodeoxyglucose positron emission tomography. *Journal of the American College of Cardiology* 2006;48(9):1825-31.
38. Figueroa AL, Abdelbaky A, Truong QA, et al. Measurement of arterial activity on routine FDG PET/CT images improves prediction of risk of future CV events. *JACC: Cardiovascular Imaging* 2013;6(12):1250-59.
39. Tawakol A, Singh P, Rudd JH, et al. Effect of treatment for 12 weeks with rilapladib, a lipoprotein-associated phospholipase A2 inhibitor, on arterial inflammation as assessed with 18F-fluorodeoxyglucose-positron emission tomography imaging. *Journal of the American College of Cardiology* 2014;63(1):86-88.
40. Bucerius J, Hyafil F, Verberne HJ, et al. Position paper of the Cardiovascular Committee of the European Association of Nuclear Medicine (EANM) on PET imaging of atherosclerosis. *European journal of nuclear medicine and molecular imaging* 2016;43(4):780-92.
41. O'Donoghue ML, Braunwald E, White HD, et al. Effect of darapladib on major coronary events after an acute coronary syndrome: the SOLID-TIMI 52 randomized clinical trial. *Jama* 2014;312(10):1006-15.
42. Schwartz GG, Olsson AG, Abt M, et al. Effects of dalcetrapib in patients with a recent acute coronary syndrome. *New England Journal of Medicine* 2012;367(22):2089-99.
43. Investigators S. Darapladib for preventing ischemic events in stable coronary heart disease. *New England Journal of Medicine* 2014;370(18):1702-11.
44. He J, Zhang X, Wei Y, et al. Low-dose interleukin-2 treatment selectively modulates CD4+ T cell subsets in patients with systemic lupus erythematosus. *Nature medicine* 2016;22(9):991-93.

For peer review only

Figure Legends

Figure 1 - Summary of Trial Design.
Figure 2 - IVORY trial visits – timeline and assessments
Figure 3 - Phenotypic Characterisation and Antibodies used in fluorescence-activated cell sorting (FACS) analysis of T cell subsets
Figure 4 - Analysis methods used to quantify vascular inflammation in the index vessel

For peer review only

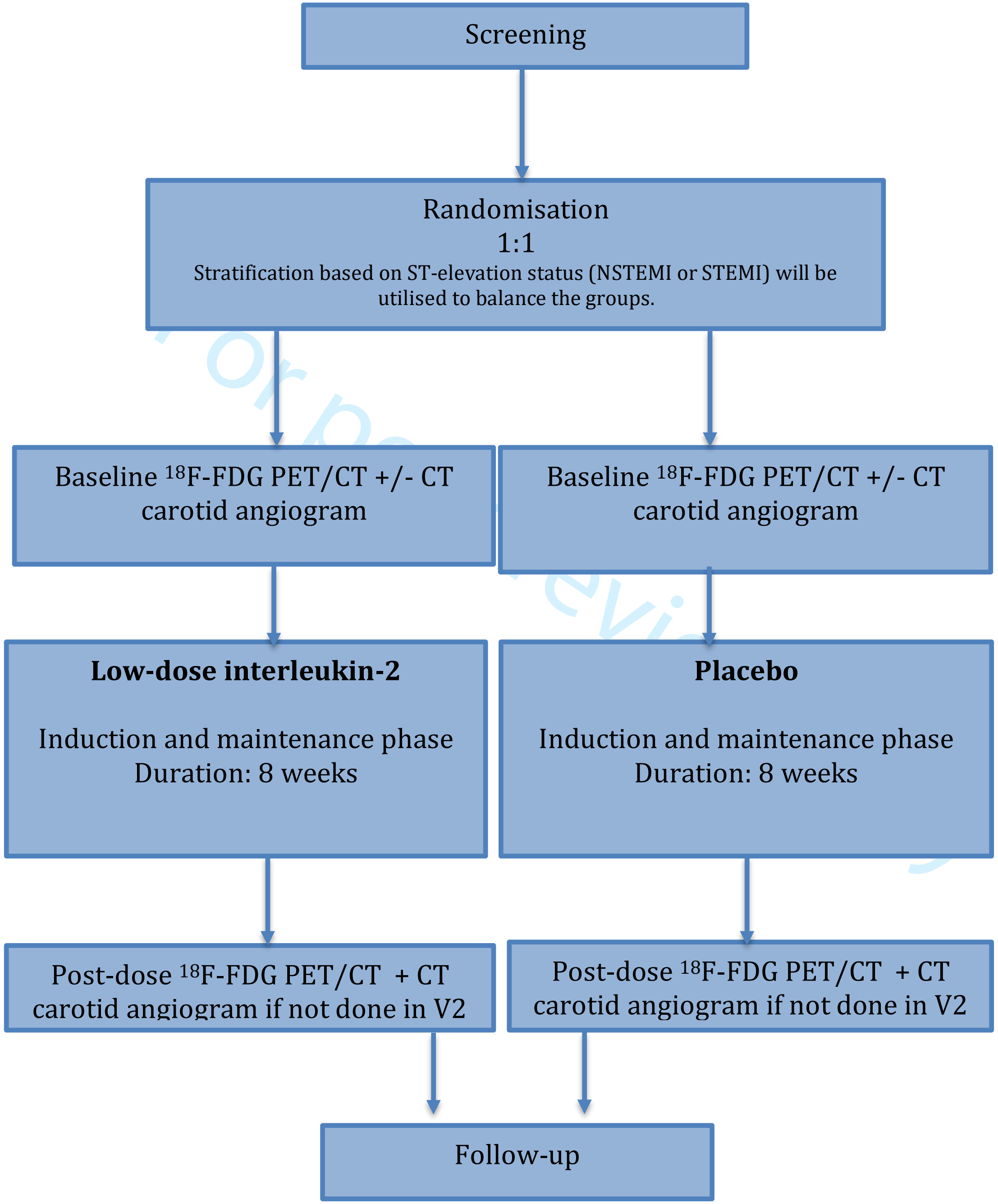

Figure 1:

Summary of Trial Design

Figure 2 : IVORY Trial timeline of visits and assessments

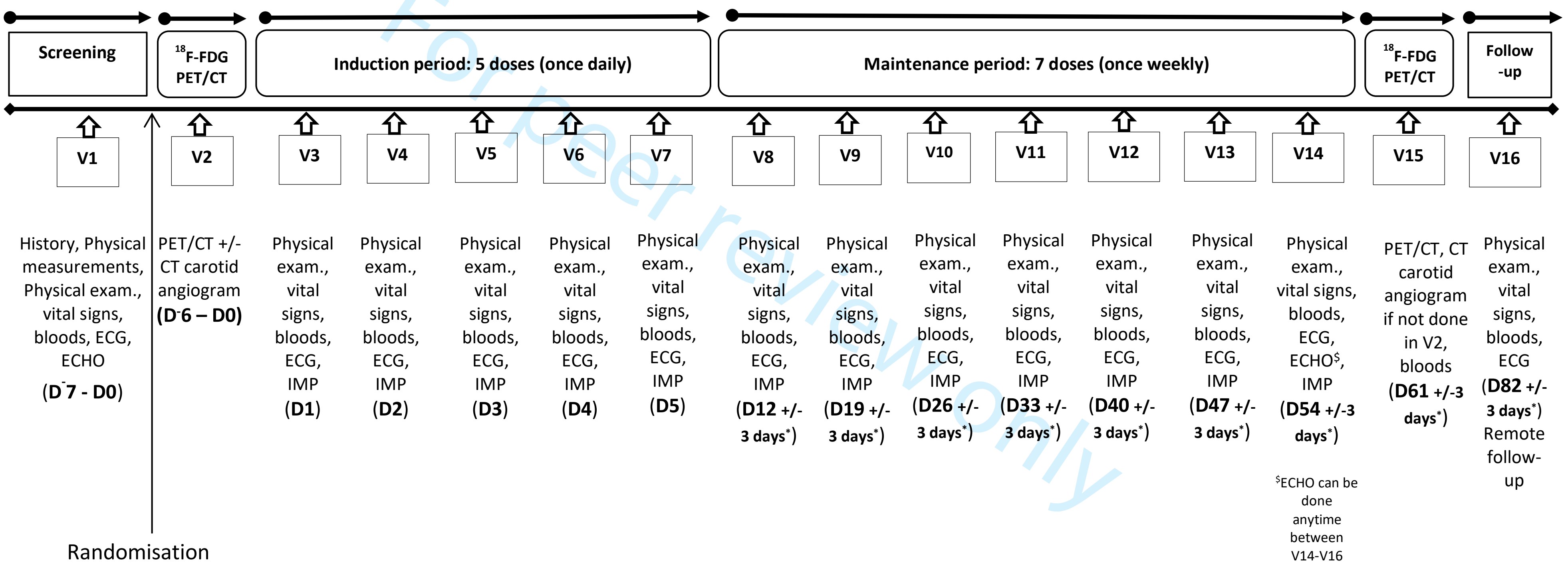

3a: Phenotypic characterization of T cell subsets by flow cytometry[44]

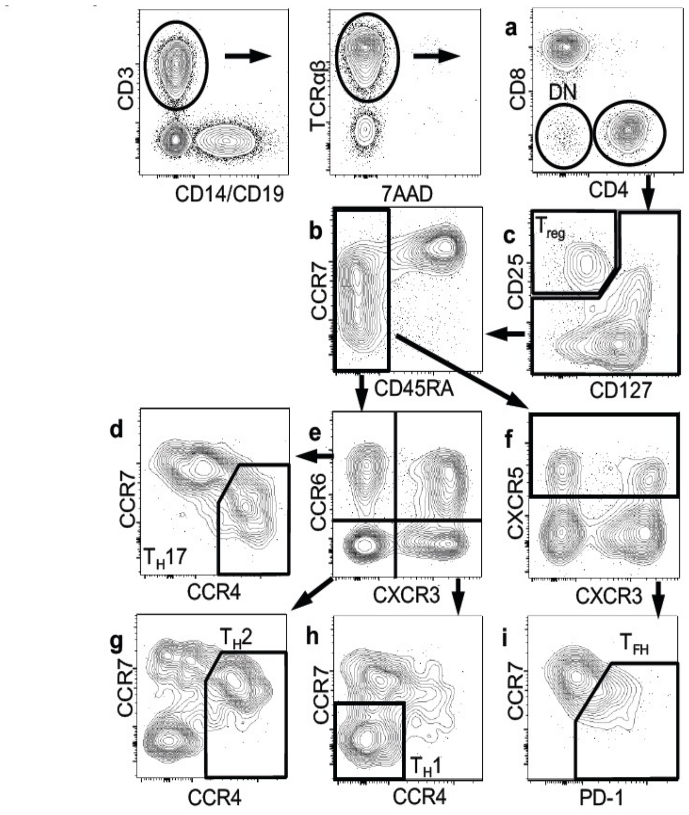

DN (CD4-CD8-)	CD4- CD8 -CD3+ TCRαβ+ CD14- CD19- 7AAD-
T_{reg}	CD25high CD127low CD4+ CD8 -CD3+ TCRαβ+ CD14- CD19- 7AAD-
T_H1	CXCR3+ CCR6- CCR4- CCR7low CD4+ CD8 -CD3+ TCRαβ+ CD14- CD19- 7AAD-
T_H2	CCR4+ CXCR3- CCR6- CCR7low CD4+ CD8 -CD3+ TCRαβ+ CD14- CD19- 7AAD-
T_H17	CCR4+ CCR6+ CXCR3- CCR7low CD4+ CD8 -CD3+ TCRαβ+ CD14- CD19- 7AAD-
T_{FH}	CXCR5+ PD-1+ CCR7low CD4+ CD8 -CD3+ TCRαβ+ CD14- CD19- 7AAD-

3b: Antibodies used in flow cytometry T cell subset analysis

Antibody	CD	BD Cat NO:
**CCR6**	CD196	**551773** BD Pharmingen™ PE Mouse Anti-Human
**CD25**	CD25	**564467** BD Horizon™ BB515 Mouse Anti-Human
**CCR4**	CD194	**566475** BD Pharmingen™ BB700 Mouse Anti-Human
**CCR7**	CD197	**557648** BD Pharmingen™ PE-Cy™7 Rat Anti-Human
**CXCR5**	CD185	**558113** BD Pharmingen™ Alexa Fluor® 647 Rat Anti-Human
**CD4**	CD4	**557922** Alexa Fluor® 700 Mouse Anti-Human
**CD45RA**	CD45RA	**560674** BD Pharmingen™ APC-H7 Mouse Anti-Human
**CXCR3**	CD183	**562558** BD Horizon™ BV421 Mouse Anti-Human
**CD3**	CD3	**563109** BV510 Mouse Anti-Human
**CD127 (IL-7Rα)**	CD127	**562662** BD Horizon™ BV605 Mouse Anti-Human
**CD8**	CD8	**563677** BD Horizon™ BV711 Mouse Anti-Human
**PD-1**	CD279	**563789** BD Horizon™ BV711 Mouse Anti-Human

Figure 4: Analysis Methods Used to Quantify Vascular Inflammation in the Index Vessel

A series of contiguous segments from the index vessel are illustrated. Atherosclerotic plaques in the vessel wall are indicated in yellow. the value within each segment represents the maximum tissue-to-background ratio (TBR) for that segment. Active segments were predefined as segments with TBR >1.6.[34]

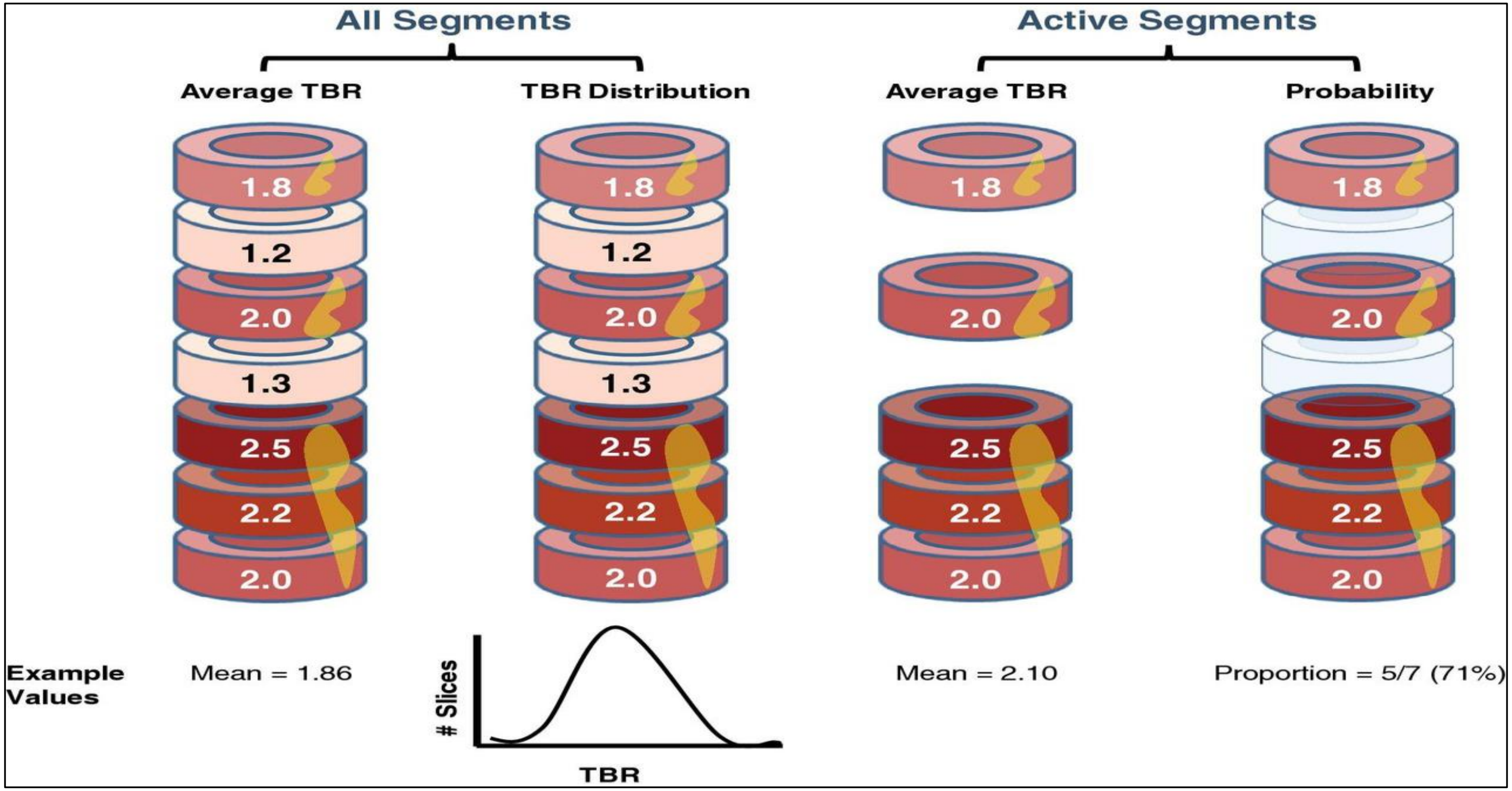

For peer review only

Supplementary Material

Supplementary Table 1 :
IVORY Inclusion and Exclusion Criteria

**Inclusion Criteria**	● Able to provide written informed consent to participate<br>● Aged between 18 and 85<br>● Current admission (on the screening visit) with an acute coronary syndrome - ST elevation myocardial infarction (STEMI), non-ST elevation myocardial infarction (NSTEMI), or unstable angina (UA) with symptoms suggestive of myocardial ischaemia lasting 10 minutes or longer with the patient at rest or with minimal effort<br>AND EITHER<br>i. elevated levels of troponin on admission<br>OR<br>ii. dynamic changes in ECG (new ST-T changes or T-wave inversion).<br><br>● Where applicable, to be included in the trial women must be<br>i) Postmenopausal (for the purposes of this trial, postmenopausal is defined as being amenorrhoeic for greater than 2 years with an appropriate clinical profile, e.g., age appropriate, history of vasomotor symptoms)<br>1. OR<br>ii) Have had a documented hysterectomy and/or bilateral oophorectomy or sterilised<br>1. OR<br>iii) Perimenopausal with a negative pregnancy test at screening (for the purposes of inclusion in this trial, perimenopausal is defined as women with an appropriate clinical profile, e.g., age appropriate, history of vasomotor symptoms, irregular periods). They will also have to comply with the use of contraception for the duration of the trial and undergo additional pregnancy tests during the trial.<br><br>● High sensitivity C-reactive protein of >2 mg/L at screening<br>● Willingness and possibility to start dosing within 14 days from initial date of admission to the primary hospital for ACS<br>● Able to comply with all trial mandated visits.
**Exclusion Criteria**	● Current presentation (at screening) with cardiogenic shock (systolic blood pressure <80 mmHg, unresponsive to fluids, or necessitating catecholamines).<br>● Current presentation with cardiac arrest<br>● Signs or symptoms of active infection requiring intravenous antibiotic treatment at screening<br>● History of malignancies requiring active treatment (however, patients with a history of treated localised basal or squamous cell skin cancer are not excluded from participation in this trial)<br>● History of solid organ transplantation or other bone marrow transplantation<br>● History of recurrent epileptic seizures in the previous 4 years; repetitive or difficult to control seizures, coma or toxic psychosis lasting >48 hours<br>● Uncontrolled hypotension (systolic blood pressure <80mmHg or diastolic blood pressure <50mmHg) OR uncontrolled hypertension (systolic blood pressure>180mmHg or diastolic blood pressure>120mmHg) at screening<br>● Average corrected QT interval (QTc) > 450msecs using Bazett's formula from average of triplicate ECGs or >480msecs if bundle branch block is present.<br>● Renal impairment defined as creatinine clearance [Cockcroft-Gault] <45ml/min at screening<br>● Liver dysfunction. Defined as alanine transaminase (ALT) >2 x upper limit of normal (ULN) at screening<br>● Evidence of cholestasis defined as elevated total bilirubin levels (TBL) > 1.5 x ULN and alkaline phosphatase (ALP) >1.5 x ULN at screening.<br>● Known hypothyroidism or hyperthyroidism<br>● Known autoimmune disease requiring active immunosuppressive treatment<br>● Any oral or intravenous immunosuppressive treatment including regular prednisolone, hydrocortisone or disease modifying drugs. [Inhaled or topical steroids are permissible]<br>● Patients on cytotoxic drugs and interferon-alpha<br>● Diabetics on oral hypoglycaemics/diet control with a haemoglobin A1c (HbA1c) diabetes control and complications assay (DCCT) > 8% OR HbA1c standardised to

international federation of clinical chemistry and laboratory medicine (IFCC) >64mmol/mol, at screening. Diabetics on insulin are excluded from the study.

- Contraindication to IL-2 treatment or hypersensitivity to IL-2 or to any of its excipients
- Participation in a previous research trial in the last 3 years which involved exposure to significant ionising radiation (i.e., cumulative research radiation dose >5mSv)
- Participation in a clinical trial where the patient has received a drug or new chemical entity within 30 days or 5 half-lives, or twice the duration of the biological effect of the drug (whichever is longer) prior to the first dose of trial medication.
- Any medical history or clinically relevant abnormality that is deemed by the principal investigator (PI)/delegate to make the patient ineligible for inclusion because of a safety concern
- Pregnant women or breast-feeding women
- Patients who are COVID-19 PCR positive at the time of screening
- Known severe allergy to the CT-contrast agents.

For peer review only

Patient Informed Consent form

Trial Title: Low-dose interleukin-2 for the reduction of vascular inflammation in Acute Coronary Syndromes (IVORY)

Principal Investigator: **Participant Number:** __________

If you agree with each sentence below, please initial the box **INITIALS**

1	I have read and understood the Participant Information Sheet version 3.0, dated 01 March 2021 for the above trial and I confirm that the trial procedures and information have been explained to me. I have had the opportunity to ask questions and I am satisfied with the answers and explanations provided.	
2	I understand that my participation in this trial is voluntary and that I am free to withdraw at any time, without giving a reason and without my medical care or legal rights being affected.	
3	I understand that personal information about me will be collected and used in accordance with this information sheet. This information will be kept in the strictest confidence and none of my personal data will be published.	
4	I understand that sections of my medical notes or information related directly to my participation in this trial may be looked at by responsible individuals from the sponsor, regulatory authorities and research personnel where it is relevant to my taking part in research and that they will keep my personal information confidential. I give permission for these individuals to have access to my records.	
5	I understand that my GP will be informed of my participation and any incidental clinical findings of relevance in this trial as well as information about the IVORY trial.	
6	I understand that my personal data might be transferred between the trial team at different trial sites in relation to my participation in this trial. I understand that any personal data will be sent using (secure/encrypted mail servers etc).	
7	I have read and understood the compensation arrangements for this trial as specified in the Participant Information Sheet.	
8	I understand that the doctors in charge of this trial may close the trial, or stop my participation in it at any time without my consent.	
9	I have read and understood my responsibilities for the trial including using appropriate contraception as listed in section 6.	
10	I understand that any data and samples already collected will still be used by the trial team if I withdraw/am withdrawn from the trial, unless I instruct the team to destroy them.	

OPTIONAL

		YES	NO
11	I agree that in the event that there are any remaining unused samples at the end of the IVORY trial analysis these may be used in other approved research studies.		
12	I give my permission to be re-contacted in the future by this research team and other research teams to invite me to take part in new studies that may be of interest. I understand that this does not oblige me to take part in further research.		

For peer review only

I agree to participate in this trial:

______________________	______________________	________
Name of patient	Signature	Date
______________________	______________________	________
Name of person taking consent	Signature	Date

Time of Consent (24hr clock) ________:________

1 copy for the patient, 1 copy for the trial team, 1 copy to be retained in the hospital notes.

For peer review only

Reporting checklist for protocol of a clinical trial.

Based on the SPIRIT guidelines.

Instructions to authors

Complete this checklist by entering the page numbers from your manuscript where readers will find each of the items listed below.

Your article may not currently address all the items on the checklist. Please modify your text to include the missing information. If you are certain that an item does not apply, please write "n/a" and provide a short explanation.

Upload your completed checklist as an extra file when you submit to a journal.

In your methods section, say that you used the SPIRITreporting guidelines, and cite them as:

Chan A-W, Tetzlaff JM, Gøtzsche PC, Altman DG, Mann H, Berlin J, Dickersin K, Hróbjartsson A, Schulz KF, Parulekar WR, Krleža-Jerić K, Laupacis A, Moher D. SPIRIT 2013 Explanation and Elaboration: Guidance for protocols of clinical trials. BMJ. 2013;346:e7586

For peer review only

		Reporting Item	Page Number
**Administrative information**			
Title	#1	Descriptive title identifying the study design, population, interventions, and, if applicable, trial acronym	1
Trial registration	#2a	Trial identifier and registry name. If not yet registered, name of intended registry	2
Trial registration: data set	#2b	All items from the World Health Organization Trial Registration Data Set	1-12
Protocol version	#3	Date and version identifier	1
Funding	#4	Sources and types of financial, material, and other support	12
Roles and responsibilities: contributorship	#5a	Names, affiliations, and roles of protocol contributors	1,12

Roles and responsibilities: sponsor contact information	#5b	Name and contact information for the trial sponsor	5,6,12
Roles and responsibilities: sponsor and funder	#5c	Role of study sponsor and funders, if any, in study design; collection, management, analysis, and interpretation of data; writing of the report; and the decision to submit the report for publication, including whether they will have ultimate authority over any of these activities	5,12
Roles and responsibilities: committees	#5d	Composition, roles, and responsibilities of the coordinating centre, steering committee, endpoint adjudication committee, data management team, and other individuals or groups overseeing the trial, if applicable (see Item 21a for data monitoring committee)	11
**Introduction**			
Background and rationale	#6a	Description of research question and justification for undertaking the trial, including summary of relevant studies (published and unpublished) examining benefits and harms for each intervention	3,4
Background and rationale: choice of comparators	#6b	Explanation for choice of comparators	3,4
Objectives	#7	Specific objectives or hypotheses	3,4,9
Trial design	#8	Description of trial design including type of trial (eg, parallel group, crossover, factorial, single group), allocation ratio, and framework (eg, superiority, equivalence, non-inferiority, exploratory)	7
**Methods: Participants, interventions, and outcomes**			
Study setting	#9	Description of study settings (eg, community clinic, academic hospital) and list of countries where data will be collected. Reference to where list of study sites can be obtained	5

For peer review only

Eligibility criteria	#10	Inclusion and exclusion criteria for participants. If applicable, eligibility criteria for study centres and individuals who will perform the interventions (eg, surgeons, psychotherapists)	5
Interventions: description	#11a	Interventions for each group with sufficient detail to allow replication, including how and when they will be administered	6-9
Interventions: modifications	#11b	Criteria for discontinuing or modifying allocated interventions for a given trial participant (eg, drug dose change in response to harms, participant request, or improving / worsening disease)	7
Interventions: adherance	#11c	Strategies to improve adherence to intervention protocols, and any procedures for monitoring adherence (eg, drug tablet return; laboratory tests)	6-9
Interventions: concomitant care	#11d	Relevant concomitant care and interventions that are permitted or prohibited during the trial	6-9
Outcomes	#12	Primary, secondary, and other outcomes, including the specific measurement variable (eg, systolic blood pressure), analysis metric (eg, change from baseline, final value, time to event), method of aggregation (eg, median, proportion), and time point for each outcome. Explanation of the clinical relevance of chosen efficacy and harm outcomes is strongly recommended	9-11
Participant timeline	#13	Time schedule of enrolment, interventions (including any run-ins and washouts), assessments, and visits for participants. A schematic diagram is highly recommended (see Figure)	6-9
Sample size	#14	Estimated number of participants needed to achieve study objectives and how it was determined, including clinical and statistical assumptions supporting any sample size calculations	5-6
Recruitment	#15	Strategies for achieving adequate participant enrolment to reach target sample size	5-7
**Methods: Assignment of interventions (for controlled trials)**			
Allocation: sequence generation	#16a	Method of generating the allocation sequence (eg, computer-generated random numbers), and list of any factors for stratification. To reduce predictability of a random sequence, details of any planned restriction (eg, blocking) should be	6

		provided in a separate document that is unavailable to those who enrol participants or assign interventions	
Allocation concealment mechanism	#16b	Mechanism of implementing the allocation sequence (eg, central telephone; sequentially numbered, opaque, sealed envelopes), describing any steps to conceal the sequence until interventions are assigned	6
Allocation: implementation	#16c	Who will generate the allocation sequence, who will enrol participants, and who will assign participants to interventions	6
Blinding (masking)	#17a	Who will be blinded after assignment to interventions (eg, trial participants, care providers, outcome assessors, data analysts), and how	6-7
Blinding (masking): emergency unblinding	#17b	If blinded, circumstances under which unblinding is permissible, and procedure for revealing a participant's allocated intervention during the trial	7
**Methods: Data collection, management, and analysis**			
Data collection plan	#18a	Plans for assessment and collection of outcome, baseline, and other trial data, including any related processes to promote data quality (eg, duplicate measurements, training of assessors) and a description of study instruments (eg, questionnaires, laboratory tests) along with their reliability and validity, if known. Reference to where data collection forms can be found, if not in the protocol	6
Data collection plan: retention	#18b	Plans to promote participant retention and complete follow-up, including list of any outcome data to be collected for participants who discontinue or deviate from intervention protocols	6
Data management	#19	Plans for data entry, coding, security, and storage, including any related processes to promote data quality (eg, double data entry; range checks for data values). Reference to where details of data management procedures can be found, if not in the protocol	6
Statistics: outcomes	#20a	Statistical methods for analysing primary and secondary outcomes. Reference to where other details of the statistical analysis plan can be found, if not in the protocol	9-11

For peer review only

Statistics: additional analyses	#20b	Methods for any additional analyses (eg, subgroup and adjusted analyses)	9-11
Statistics: analysis population and missing data	#20c	Definition of analysis population relating to protocol non-adherence (eg, as randomised analysis), and any statistical methods to handle missing data (eg, multiple imputation)	9-11
**Methods: Monitoring**			
Data monitoring: formal committee	#21a	Composition of data monitoring committee (DMC); summary of its role and reporting structure; statement of whether it is independent from the sponsor and competing interests; and reference to where further details about its charter can be found, if not in the protocol. Alternatively, an explanation of why a DMC is not needed	11
Data monitoring: interim analysis	#21b	Description of any interim analyses and stopping guidelines, including who will have access to these interim results and make the final decision to terminate the trial	11
Harms	#22	Plans for collecting, assessing, reporting, and managing solicited and spontaneously reported adverse events and other unintended effects of trial interventions or trial conduct	11
Auditing	#23	Frequency and procedures for auditing trial conduct, if any, and whether the process will be independent from investigators and the sponsor	5-6
**Ethics and dissemination**			
Research ethics approval	#24	Plans for seeking research ethics committee / institutional review board (REC / IRB) approval	1
Protocol amendments	#25	Plans for communicating important protocol modifications (eg, changes to eligibility criteria, outcomes, analyses) to relevant parties (eg, investigators, REC / IRBs, trial participants, trial registries, journals, regulators)	4,11
Consent or assent	#26a	Who will obtain informed consent or assent from potential trial participants or authorised surrogates, and how (see Item 32)	6

For peer review only

Consent or assent: ancillary studies	#26b	Additional consent provisions for collection and use of participant data and biological specimens in ancillary studies, if applicable	6
Confidentiality	#27	How personal information about potential and enrolled participants will be collected, shared, and maintained in order to protect confidentiality before, during, and after the trial	6
Declaration of interests	#28	Financial and other competing interests for principal investigators for the overall trial and each study site	11
Data access	#29	Statement of who will have access to the final trial dataset, and disclosure of contractual agreements that limit such access for investigators	1,11
Ancillary and post trial care	#30	Provisions, if any, for ancillary and post-trial care, and for compensation to those who suffer harm from trial participation	6
Dissemination policy: trial results	#31a	Plans for investigators and sponsor to communicate trial results to participants, healthcare professionals, the public, and other relevant groups (eg, via publication, reporting in results databases, or other data sharing arrangements), including any publication restrictions	1
Dissemination policy: authorship	#31b	Authorship eligibility guidelines and any intended use of professional writers	11
Dissemination policy: reproducible research	#31c	Plans, if any, for granting public access to the full protocol, participant-level dataset, and statistical code	1,11
**Appendices**			
Informed consent materials	#32	Model consent form and other related documentation given to participants and authorised surrogates	6
Biological specimens	#33	Plans for collection, laboratory evaluation, and storage of biological specimens for genetic or molecular analysis in the current trial and for future use in ancillary studies, if applicable	6

The SPIRIT Explanation and Elaboration paper is distributed under the terms of the Creative Commons Attribution License CC-BY-NC. This checklist was completed on 02. March 2022 using https://www.goodreports.org/, a tool made by the EQUATOR Network in collaboration with Penelope.ai

For peer review only

BMJ Open

Low-dose interleukin-2 for the reduction of vascular inflammation in acute coronary syndromes (IVORY): protocol and study rationale for a randomised, double-blind, placebo-controlled, phase II clinical trial

Journal:	*BMJ Open*
Manuscript ID	bmjopen-2022-062602.R1
Article Type:	Protocol
Date Submitted by the Author:	18-Jul-2022

Complete List of Authors:	Sriranjan, Rouchelle; University of Cambridge, Department of Medicine, Division of cardiovascular medicine<br>Zhao, Tian; University of Cambridge Medicine, Division of Cardiovascular Medicine<br>Tarkin, Jason; University of Cambridge, Department of Medicine, Division of Cardiovascular Medicine<br>Hubsch, Annette; University of Cambridge, Department of Medicine, Division of Experimental Medicine and Immunotherapeutics (EMIT)<br>Helmy, Joanna; University of Cambridge Medicine, Experimental Medicine and Immunotherapeutics (EMIT) Division<br>Vamvaka, Evangelia; University of Cambridge, Department of Medicine, Division of Experimental Medicine and Immunotherapeutics (EMIT)<br>Jalaludeen, Navazh; University of Cambridge, Department of Medicine, Division of Experimental Medicine and Immunotherapeutics (EMIT)<br>Bond, Simon; Cambridge University Hospitals NHS Foundation Trust, Cambridge Clinical Trials Unit<br>Hoole, Stephen; Papworth Hospital NHS Foundation Trust, Cardiology<br>Knott, Philip; Cambridge University Hospitals NHS Foundation Trust, Department of Clinical Immunology<br>Buckenham, Samantha; Cambridge University Hospitals NHS Foundation Trust, Department of Immunology<br>Warnes, Victoria; Cambridge University Hospitals NHS Foundation Trust, Department of Nuclear Medicine<br>Bird, Nick; Cambridge University Hospitals NHS Foundation Trust<br>Cheow, Heok; Cambridge University Hospitals NHS Foundation Trust, Department of Nuclear Medicine<br>Templin, Heike; Cambridge University Hospitals NHS Foundation Trust, Cambridge Clinical Trials Unit<br>Cacciottolo, Paul; University of Cambridge, Department of Medicine,Division of Experimental Medicine and Immunotherapeutics (EMIT)<br>Rudd, James; University of Cambridge Medicine, Division of Cardiovascular Medicine<br>Mallat, Ziad; University of Cambridge Medicine, Division of Cardiovascular Medicine<br>Cheriyan, Joseph; University of Cambridge Medicine, Division of Experimental Medicine & Immunotherapeutics

**Primary Subject Heading**:	Cardiovascular medicine
Secondary Subject Heading:	Cardiovascular medicine, Immunology (including allergy), Pharmacology and therapeutics
Keywords:	Cardiology < INTERNAL MEDICINE, Cardiovascular imaging < RADIOLOGY & IMAGING, Immunology < NATURAL SCIENCE DISCIPLINES, Clinical trials < THERAPEUTICS

SCHOLARONE™ Manuscripts

Low-dose interleukin-2 for the reduction of vascular inflammation in acute coronary syndromes (IVORY): protocol and study rationale for a randomised, double-blind, placebo-controlled, phase II clinical trial

Rouchelle Sriranjan[1], Tian Zhao[1], Jason Tarkin[1], Annette Hubsch[2], Joanna Helmy[2], Evangelia Vamvanka[2], Navazh Jalaludeen[2], Simon Bond[3], Stephen Hoole[4], Philip Knott[5], Samantha Buckenham[5], Victoria Warnes[6], Nick Bird[6], Heok Cheow[6], Heike Templin[3], Paul Cacciottolo[2], James H.F. Rudd[1]*, Ziad Mallat[1]*, Joseph Cheriyan[2,3]*

***Joint senior authors**

Correspondence to:

Dr Rouchelle Sriranjan
Department of Medicine
Division of Cardiovascular Medicine
University of Cambridge Medicine, Cambridge, UK
Addenbrooke's Hospital
Hills Road
CAMBRIDGE
CB2 2QQ
rfss2@cam.ac.uk

Dr Joseph Cheriyan
The Experimental Medicine & Immunotherapeutic Unit
Level 3, Addenbrooke's Centre for Clinical Investigation (ACCI)
Box 110
Addenbrooke's Hospital
Hills Road
CAMBRIDGE
CB2 2QQ
jc403@medschl.cam.ac.uk

Author affiliations:
[1]Department of Medicine, Division of Cardiovascular Medicine, University of Cambridge, Cambridge, UK
[2]Division of Experimental Medicine and Immunotherapeutics (EMIT), Department of Medicine, University of Cambridge, Cambridge, UK
[3]Cambridge Clinical Trials Unit, Cambridge University Hospitals, Cambridge, UK
[4]Department of Interventional Cardiology, Royal Papworth Hospital NHS Trust, Cambridge, UK
[5]Department of Immunology, Cambridge University Hospitals, Cambridge, UK
[6]Department of Nuclear Medicine, Cambridge University Hospitals, Cambridge, UK

Word count: 3845

For peer review only

ABSTRACT

Introduction: Inflammation plays a critical role in the pathogenesis of atherosclerosis, the leading cause of ischemic heart disease (IHD). Studies in pre-clinical models have demonstrated that an increase in regulatory T cells (Tregs), which have a potent immune modulatory action, led to a regression of atherosclerosis. The Low-dose InterLeukin-2 (IL-2) in patients with stable ischaemic heart disease and Acute Coronary Syndromes (LILACS) study, established the safety of low-dose IL-2 and its biological efficacy in IHD. The IVORY trial is designed to assess the effects of low-dose IL-2 on vascular inflammation in patients with acute coronary syndromes (ACS).

Methods and analysis: In this study, we hypothesize that low-dose IL-2 will reduce vascular inflammation in patients presenting with ACS. This is a double-blind, randomised, placebo-controlled, phase II clinical trial. Patients will be recruited across two centres, a district general hospital and a tertiary cardiac centre in Cambridge, UK. Sixty ACS (unstable angina, non-ST elevation, or ST elevation myocardial infarction) patients with high sensitivity CRP (hsCRP) levels >2mg/L, will be randomised to receive either 1.5 x 10^6IU of low-dose IL-2 or placebo (1:1). Dosing will commence within 14 days of admission. Dosing will comprise of an induction and a maintenance phase. 2-deoxy-2-[fluorine-18] fluoro-D-glucose (^{18}F-FDG) positron emission tomography/ computed tomography (PET/CT) scans will be performed before and after dosing. The primary endpoint is the change in mean maximum target to background ratios (TBR_{max}) in the index vessel between baseline and follow-up scans. Changes in circulating T cell subsets will be measured as secondary endpoints of the study. The safety and tolerability of extended dosing with low-dose IL-2 in patients with ACS will be evaluated throughout the study.

Ethics and dissemination: The Health Research Authority and Health and Care Research Wales, UK (19/YH/0171), approved the study. Written informed consent is required to participate in the trial. The results will be reported through peer-reviewed journals and conference presentations.

Trial registration number: ClinicalTrials.gov, NCT04241601. Protocol date: 01.03.2021, version 3.0, approval 09.04.2021.

For peer review only

Strengths and limitations of this study

- **This study explores the clinical efficacy of low-dose IL-2 in reducing vascular inflammation in patients acutely presenting with ACS, using a well validated imaging biomarker (^{18}F-FDG).**
- **Changes that occur in a wide range of circulating lymphocyte subsets with extended dosing with low-dose IL-2 will be studied in the acute ACS patient population at multiple timepoints.**
- **The safety of extended dosing with low-dose IL-2 in patients acutely presenting with ACS will be studied in this trial, using multiple sources.**
- **Due to the early phase nature of this study, it is not powered to determine if treatment with low dose IL-2 would lead to improvements in clinical outcomes.**

INTRODUCTION

Atherosclerosis and inflammation

Inflammation plays a pivotal role in the initiation and progression of atherosclerosis, which accounts for an overwhelming majority of cardiovascular events. The role of the immune system in the pathophysiology of atherosclerosis and plaque instability is multi-faceted and orchestrated by both, the innate and adaptive components of the immune system.[1] The Canakinumab ANti-inflammatory Thrombosis Outcomes Study (CANTOS) reported for the first time that anti-inflammatory therapy significantly reduced recurrent cardiovascular events in patients with IHD and ongoing inflammation with persistently raised hsCRP levels.[2] Further efforts to harness the immune system for pharmacological therapeutic strategies in cardiovascular disease (CVD) are currently underway.[3 4]

Due to their critical role in mediating immune tolerance and anti-atherosclerotic effects, natural and inducible Treg cells ($CD4^{+}CD25^{+}FOPX3$) are a focus of novel therapeutic strategies. Initial studies in pre-clinical models showed that Tregs reduced plaque inflammation, slowed progression and even led to regression of atherosclerosis.[5 6] Tregs have also been implicated in moderating the post-ischaemic immune responses triggered by self-antigens presented by necrotic myocardium.[7] In line with this finding, it has also been demonstrated that Tregs promote myocardial wound healing, attenuate adverse left ventricular remodelling and improve cardiac function. [8-11] The Malmo Diet and Cancer study further demonstrated an increased risk of acute coronary events in patients with low levels of baseline circulating Treg cells.[12] Observational studies have also shown that patients with ACS have lower numbers of circulating Tregs with impaired function.[13-16] Furthermore, low levels of Tregs maybe also be associated with plaque instability.[15]

Interleukin-2 (IL-2) plays a crucial role in immune homeostasis by determining the balance between T effector (Teffs) and Tregs.[17 18] Whilst IL-2 is implicated in the development of both Teffs and Tregs, unlike Teffs, Treg cells constitutively express an IL-2 receptor (IL-2Rα) which binds to IL-2 with high affinity, making this subset very sensitive to IL-2.[19] Of the cytokines involved in Treg development and stability, IL-2 is the most essential[20].

For peer review only

The administration of low dose IL-2 has been used in autoimmune conditions such as graft versus host disease (GVHD), autoimmune vasculitis, hepatitis, type 1 diabetes mellitus (T1DM), and systemic lupus erythematosus (SLE), to boost Treg numbers with promising results.[17 21-24] Furthermore, a recent study involving patients with 11 auto-immune conditions demonstrated the clinical efficacy and safety of low-dose IL-2.[25]

LILACS

The Low dose InterLeukin-2 in patients with stable ischaemic heart disease and Acute Coronary Syndromes (LILACS) was the first study to explore the safety of low dose of IL-2 and its ability to expand Treg cells in this patient cohort. This was a double blinded, randomised controlled adaptive phase I/IIa trial.[26] Twenty-five patients with stable IHD were dosed in part A with aldesleukin (recombinant IL-2). The dose range used in this trial was between 0.3-3 x 10^6IU. The ratio of IL-2 to placebo was 3:2 for each group. An increase in Treg cells was observed at the doses tested without any significant increases in Teff cells.[26] Part B, which included patients with ACS (non-ST elevation myocardial infarction and unstable angina), was carried out upon completion of part A. Two doses (1.5 and 2.5 x 10^6IU) were used in part B (IL-2: placebo = 6:2). In both parts, the low-dose IL-2 and placebo were administered for 5 consecutive days. Low-dose IL-2 was well tolerated in both stable IHD and ACS patients. The most commonly occurring adverse reactions were injection site reactions, which were temporary. Other adverse reactions included flu like symptoms and myalgia. No serious adverse events (SAEs) were observed in part A. Two SAEs were noted in part B that were deemed to be unrelated to the investigational medical product (IMP). In LILACS, the target dose needed to achieve a 75% increase in Tregs was determined as 1.46 x10^6IU.

Based on the promising results of LILACS, we hypothesized that low-dose IL-2 would reduce vascular inflammation in patients with ACS as assessed by ^{18}F-FDG PET/CT imaging before and after treatment.

^{18}F-FDG is the most widely used radionuclide in imaging inflammation in the context of atherosclerosis. Limitations of ^{18}F-FDG include the fact that it cannot be used to quantify inflammation in coronary arteries reliably and that the ^{18}F-FDG signal is not inflammatory cell specific as all glucose metabolising cells take up ^{18}F-FDG.[27-29] However, with respect to atherosclerosis, *ex vivo* histological studies from arterial specimens have shown a strong correlation between ^{18}F-FDG uptake and macrophage density.[30 31] This is thought to be due to increased glucose turnover in macrophages compared to other cells within the atherosclerotic plaque. Furthermore ^{18}F-FDG quantification is highly reliable and reproducible in large to medium sized vessels.[32] ^{18}F-FDG PET/CT has been used by our group and others as a biomarker for drug development in Phase II cardiovascular drug intervention studies.[33-35] ^{18}F-FDG PET/CT has specifically been used to study the effect of statins and anti-inflammatory compounds on vascular inflammation.[36-40]

METHODS AND ANALYSIS

For peer review only

The IVORY trial is a prospective, randomised, double-blind, placebo-controlled, Phase II clinical trial. This is an academic study involving two centres namely, Cambridge University Hospitals (CUH) and Royal Papworth Hospital (RPH), in Cambridge, UK. Overall study coordination, monitoring and auditing will be undertaken by the Cardiovascular Trials Office of the Cambridge Clinical Trials Unit (CCTU), CUH. The study is sponsored by CUH.

Study population

To be included, participants must be admitted with a diagnosis of ACS. The study will include patients presenting with ST elevation myocardial infarction (STEMI), non-ST elevation myocardial infarction (NSTEMI), or unstable angina (UA). The participants should have symptoms suggestive of myocardial ischaemia lasting 10 minutes or longer at rest or on minimal effort; and either have elevated levels of troponin on admission and/or dynamic electrocardiogram (ECG) changes suggestive of ischaemia. Furthermore, to be included participants will require hsCRP levels >2mg/L at screening. We hypothesize patients presenting with hsCRP levels >2mg/L have residual systemic inflammation and are at higher risk of recurrent cardiovascular events. Therefore, recruitment will be restricted to these patients as they are most likely to benefit from this treatment.

Due to the ionising radiation associated with ^{18}F-FDG PET/CT scans, to be included in the trial, women must be either postmenopausal, have had a documented hysterectomy and/or bilateral oophorectomy or sterilisation procedure, or be perimenopausal with a negative pregnancy test at screening. Perimenopausal women will also have to comply with the use of contraception for the duration of the trial and undergo additional pregnancy tests during and after treatment.

Exclusion criteria include patients who present with cardiogenic shock, cardiac arrest, and uncontrolled hypertension/hypotension. Blood tests will be carried out to exclude patients with thyroid, severe renal, and hepatic dysfunction as specified in the protocol. Patients with active infection, history of malignancy requiring active treatment or on oral/intravenous immune suppression therapy will also be excluded. Patients with type 1, or type 2 diabetes mellitus (T2DM) with poor control, will be excluded due to the challenges in analysing ^{18}F-FDG PET/CT scans in this patient cohort.[40] Due to the ongoing pandemic and to minimise potential risk, patients who are coronavirus disease 2019 (COVID-19) positive on polymerase chain reaction (PCR) at the time of screening will not be recruited to the trial. Detailed inclusion and exclusion criteria are listed in supplementary table 1.

We plan to recruit sixty patients for the IVORY study. The sample size is based on an absolute difference of 0.2 in the primary endpoint (mean TBR_{max} in the index vessel) between placebo and active treatment at the end of the dosing period. This is equivalent to a 10% difference from a reference value of 2.02 and equivalent to the size effect observed after atheroprotective therapy.[35-37] This is based on the results of previous studies, that have reported less than a 10% difference in mean TBR_{max} in the index vessel between active treatment and placebo and failed to reduce CV outcomes[34 39 41-43].

For peer review only

Assuming a SD of 0.24, 24 patients per arm, testing at 2-sided 5% significance level, would provide 80% power. Therefore, a sample size of 30 completed patients per arm was selected to account for participant dropout and poor-quality scans deemed to be unsuitable for analysis (e.g., because of motion artifact).

The study opened on 26/08/2020. Thirty-three participants have completed the trial to date, and we expect recruitment to be completed by December 2023, after which the data analysis will commence.

Dose selection for ivory

A dose of 1.5 x 10^6IU was selected for the study based on analysis of data from the LILACS trial. In ACS patients dosed in Part B of the LILACS trial, a dose of 1.5 x 10^6IU led to an 80% increase in Tregs after five consecutive days of treatment. No significant increases in Teff cells were noted at this dose.

Active drug description

Aldesleukin is produced by recombinant DNA technology using an *Escherichia coli* strain that contains a genetically engineered modification of the human IL-2 gene. Commercially available aldesleukin with an UK marketing authorisation will be used and initially prepared as per the manufacturer's guidance. This preparation will be diluted further using 5% glucose, to give a final concentration of 3.3 x 10^6 IU/ml. The administration of aldesleukin will be by subcutaneous (S/C) injection.

Placebo description

Commercially available dextrose 5% with an UK marketing authorisation at equivalent dose volume will be used for the placebo formulation.

Study protocol

The trial will be performed in accordance with the spirit and the letter of the declaration of Helsinki, the conditions and principles of Good Clinical Practice, the protocol and applicable local regulatory requirements and law. Participants will be provided with an information sheet and given the opportunity to consider the information for at least for 24 hours. They will have an opportunity during this time to discuss the study with trial staff and have any queries answered before consenting to participate in the trial. Full informed consent (supplementary file) will be taken at the screening visit (V1) by the study team. The screening visit will also include baseline blood tests, a physical examination, vitals (blood pressure, heart rate, temperature), a clinical history, and ECGs in triplicate. The screening bloods will include safety blood tests (full blood count; electrolytes/urea/creatinine; liver function tests), thyroid function tests, hsCRP, serum/urine pregnancy test where applicable and a full lipid profile.

Each participant who passes screening will be assigned an ID number. The research team will complete a case record form (CRF) for each encounter with the participant. The data is then sent to the CCTU for entry into the electronic trial database. All CRFs will be stored safely, and patient identifiable information is kept confidential.

Randomisation will then be carried out using sealed envelope

For peer review only

(www.sealedenvelope.com). Randomisation will be stratified by ST elevation status to minimise bias between the groups since ST-elevation MI patients typically have higher circulating hsCRP levels.

During the trial, the patient facing trial team, patients, and data analysis teams (immunology sample analysers and image analysers) are blinded to the treatment allocation. To maintain the overall quality and legitimacy of the clinical trial, unblinding will only occur in exceptional circumstances when knowledge of the actual treatment is essential for further clinical management of the patient.

The trial design is summarised in Figure 1. After randomisation, patients will undergo an ^{18}F-FDG PET/CT scan covering the ascending aorta and carotid arteries (V2). The ^{18}F-FDG PET/CT scans will be carried out in CUH. Patients would be instructed to fast for 6 hours prior to scanning. A finger prick blood sugar test will be performed prior to the PET/CT scan. A carotid angiogram will be undertaken after the PET/CT scan where possible. The carotid angiogram can be performed either in visit 2 or 15, as this is for anatomical correlation to enable more accurate measurement of ^{18}F-FDG uptake in the carotid arteries. A transthoracic echocardiogram will also be undertaken prior to dosing to assess the left ventricular function and regional wall motion abnormalities.

Dosing will commence within 14 days of admission, and after the baseline ^{18}F-FDG PET/CT scan (Figure 2). Dosing will consist of two phases spanning a period of 8 weeks. The induction phase will involve once daily S/C injections of IMP for 5 days (V3-V7). During the maintenance phase participants will receive once weekly S/C injections of IMP for 7 weeks. Dosing visits will last up to 1 hour during the induction phase. At each visit, participants will be screened for potential side-effects and their medications will be reviewed by a clinician. Participants will also undergo a physical examination, have their vital signs measured, have ECGs recorded, and safety bloods taken. Circulating systemic inflammatory biomarkers (hsCRP, interleukin 6), cardiac troponin I and lymphocyte subsets will be measured on V3 and V7.

During the maintenance phase (V8-V14), participants will be dosed once weekly. These visits will last 1-1.5 hours. They will be similar to the induction phase visits; however, administration of the IMP will only occur once safety bloods taken during the visit are checked. Cardiac biomarkers and lymphocyte subsets will be measured during alternate maintenance visits (V8, V10, V12, V14). Within approximately 1 week from the completion of dosing, a second post-dose ^{18}F-FDG PET/CT scan will be undertaken. The final encounter will take place approximately 4 weeks after the final dosing visit via telephone unless an in-person visit is necessary for clinical or safety reasons.

Patients will be withdrawn from the trial after recruitment if any of the pre-specified general, cardiac, renal, or liver withdrawal criteria are met. They will also be withdrawn from the trial if they are positive for COVID-19 during dosing. The withdrawal criteria are summarised in Table 1.

Table 1. IVORY study withdrawal criteria

**General**	• Cardiorespiratory arrest

	• Failure to attend 2 scheduled dosing appointments without adequate reason based on the PI assessment<br>• Severe hypersensitivity reactions will preclude any further IMP administration<br>• New seizure activity /Coma<br>• Severe lethargy or somnolence<br>• Respiratory insufficiency requiring intubation<br>• Pregnancy<br>• Withdrawal of consent<br>• PI discretion<br>• Any serious adverse reaction or adverse reaction which is deemed by investigators to be Severe AR.<br>• Any significant incidental finding on PET/CT scan clinical governance reports, which in the opinion of the PI, necessitates further investigation and management.<br>• Any medical history, clinically relevant abnormality or reason that is deemed by the PI to make the patient ineligible to continue the trial.
**Cardiac**	• In patients without left bundle branch block (LBBB) QTc interval >500ms OR change from baseline: QTcB >60ms<br>• In patients with LBBB if baseline corrected QT interval increases from <450ms to >500ms OR from 450-480ms to >530ms. (Withdrawal of patients is to be based on an average QTc value of triplicate ECGs)<br>• Patients who will be scheduled to undergo coronary artery bypass grafting<br>• Patients who develop new onset severe pulmonary oedema/new severe congestive heart failure, requiring high dose > 240 mg over 24 hours IV furosemide during admission<br>• Patients with symptomatic uncontrolled systolic BP <80 mmHg and/or diastolic BP <50 mmHg (after at least 2 repeat recordings), or severe hypertension (as defined by BP > 180 mmHg systolic BP or >120 mmHg diastolic BP on at least two readings)<br>• Patients with sustained (>30sec) or symptomatic ventricular tachycardia and patients with ventricular fibrillation will be withdrawn
**Renal**	• Serum creatinine elevation > 2 x baseline screening visit (V1)
**Liver**	• ALT ≥3x ULN and total bilirubin ≥2xULN<br>• ALT ≥5xULN.<br>• ALT ≥3xULN if associated with symptoms (new or worsening) believed to be related to hepatitis (such as fatigue, nausea, vomiting, right upper quadrant pain or tenderness or jaundice) or hypersensitivity (such as fever, rash, or eosinophilia).<br>• Isolated ALT ≥3xULN that persists for ≥4 weeks

Study procedures

a) ^{18}F-FDG scans

A General Electric Discovery 690 PET/CT scanner (Milwaukee, Wisconsin) or equivalent scanner, will be used for imaging. This will be performed using reproducible, validated methods for image acquisition, reconstruction and interpretation as recommended by the European Association of Nuclear Medicine for the conduct of clinical trials using ^{18}F-FDG PET/CT.[40] A dose of approximately 240Mbq ^{18}F-FDG will be injected through a peripheral venous cannula. The ascending aorta will be imaged 90 minutes after the ^{18}F-FDG injection. Attenuation correction and non-contrast CT scans of the ascending aorta will initially be performed. This will be followed by a single bed PET scan acquired in 3D list mode for 10 minutes, with the superior portion of the aortic arch as the upper anatomical landmark of the scan. Carotid artery imaging will be undertaken immediately after the PET/CT scans of the ascending

aorta. A single bed PET scan will be acquired in 3D list mode for 15 minutes, with the external auditory meatus as the upper anatomical landmark of the scan. PET data for the ascending aorta and carotids will be reconstructed using iterative 3D time of flight ordered-subset expectation maximization with standard corrections applied ± point-spread function modelling to reduce partial volume error. A CT carotid angiogram will also be carried out after the PET acquisition either in visit 2 or visit 15.

All scans will be anonymised. The anonymised scans will be analysed in batches of 20 by the research team. Second person checks will also be performed on a subset of these scans.

b) Lymphocyte subset assays

Lymphocyte subset assays will be performed at the Department of Clinical Immunology, CUH and this will occur within 4 hours of sample collection. The clinical investigators and laboratory technicians will be blinded to treatment allocation.

Whole blood will be assessed by performing fluorescence-activated cell sorting (FACS) to measure the absolute and percentage values for lymphocytes subsets (Teffs, T helper 1, T helper 2, T helper 17 and T follicular helper cells) and Treg cells. 50µl antibody reagent and 50µl whole blood will be added directly to the BD Trucount tube. The lyophilized pellet in the tube dissolves, releasing a known number of fluorescent beads. During analysis, the absolute number (cells/µL) of positive cells in the sample can be determined by comparing cellular events to bead events. Figure 3 summarises the phenotypic characterisation of T lymphocyte subsets and the antibodies used in the FACS analysis.[44]

Outcome measures and analysis

Image analysis

The table above lists the endpoints for this study (Table 2). The primary endpoint in this study will be the change in vascular inflammation as measured by mean TBR_{max} in the index vessel on ^{18}F-FDG PET/CT scans from baseline to follow up. This will be calculated by demarcating a region of interest (ROI) including arterial wall and lumen on each axial slice of artery (ascending aorta and both carotid arteries) on the co-registered PET/CT scan and maximum standardised uptake values (SUVmax) recorded. Subsequently, ROIs will be normalised by the blood pool ^{18}F-FDG concentration in the superior vena cava or jugular vein (for carotids) and averaged to yield an arterial mean TBR_{max} as a quantitative measure of arterial tracer uptake. The "index vessel" defined as the arterial territory with the highest mean TBR_{max} at baseline will be the primary outcome variable.[40] The change in mean TBR_{max} will be calculated for each index vessel from baseline to follow-up and compared between the treatment groups.

Table 2. IVORY study endpoint

**Primary endpoints**	• Change in vascular inflammation (as measured by mean TBR_{max} in the index vessel) on ^{18}F-FDG PET/CT from baseline to follow up scans
**Secondary endpoints**	• Change in mean TBR_{max} in each arterial region individually restricted to those slices with TBR>1.6<br>• Change in lymphocyte subsets between low dose IL-2 and placebo.

	• Change in percentage of Treg cells between low dose IL-2 and placebo.<br>• The safety and tolerability of extended dosing of IL-2 in ACS patients will be evaluated
**Exploratory Endpoints**	• The change in serum cardiac biomarkers (e.g., hsCRP, troponin, Interleukin-6).<br>• Change in ejection fraction as measured on transthoracic echocardiograms<br>• Change in phenotype and function of peripheral blood mononuclear cell (PBMC) subsets (such as B lymphocytes and Natural Killer cells) as assessed by flow cytometry, gene expression, and in vitro activation and suppression assays.

Furthermore, an analysis of change from baseline in average TBR_{max} for active segments within the index vessel will be carried out, as previously described (Figure 4).[35] An active segment will be defined as a segment of a vessel with a TBR of ≥1.6. An analysis of the probability of a segment being active within the index vessel will also be calculated.

Change from baseline in average TBR_{max} will be analysed using analysis of covariance, fitting treatment as a fixed effect, and including baseline value and ST-elevation status as covariates. Point estimates and corresponding 95% confidence intervals (CI) will be constructed with P values for the relevant comparisons of interest. All nominal p-values will be reported. Multiple testing across both analyses will be controlled using a gate-keeping approach. Multiple testing corrections will be carried out to obtain the p-values.

Average mean TBR_{max} data from the final imaging visit will be plotted to show the distribution from all segments from all index vessels within each treatment group. The difference between low-dose IL-2 and placebo will be calculated and tested using a nonparametric permutation test at an individual patient level.

The number of active segments and the total number of segments will be included in logistic regression analyses to model the probability of a segment being active. For baseline correction within each group, a model will be fitted with terms for treatment and visit. For placebo and baseline correction, a model will be fitted with treatment term, including the baseline proportion of active segments as a covariate.

Lymphocyte subset and biomarker analysis

A detailed lymphocyte subset analysis will be performed to determine the change in percentage of Treg cells ($CD3^{+}$ $CD4^{+}$ $CD8^{-}$ $CD25^{high}$ $CD127^{low}$) between low dose IL-2 and placebo throughout the treatment period using flow cytometry. Other lymphocyte subsets will also be assessed in a similar manner.

The change in serum cardiac biomarkers will be evaluated by blood sample analysis at various time points in the trial. These will include high sensitivity troponin I (hs-Trop I), hsCRP and IL-6. HsCRP at screening and all hs-Trop I measurements will be analysed by the Department of Biochemistry/Clinical Immunology at CUH. All other biomarkers will be analysed by the Core Biochemical Assay laboratory, Cambridge.

For peer review only

Categorical variables will be reported as percentages and counts. All continuous variables will be reported as mean, median, SD, minimum and maximum values. The full analysis population will include all patients who received the trial drug or placebo and completed the treatment course after randomisation.

Adverse event (ae) reporting

Safety and tolerability of low-dose IL-2 in the ACS population will be monitored throughout the course of the study. All AEs will be documented, and a medical doctor blinded to the treatment allocation will determine the causality of each in conjunction with the trial Principal Investigator. AEs, adverse reactions (ARs), serious AE/ARs (SAEs/SARs) and suspected unexpected SARs will be defined as per the International Conference on Harmonisation definitions. SAEs, serious adverse reactions (SARs), suspected unexpected serious adverse reactions (SUSARs) will be subject to expedited reporting as per the Medicines and Healthcare products Regulatory Agency (MHRA) guidelines. Severe ARs and SARs will also be documented on a separate log, as these are part of the trial stopping criteria. For all AEs, an assessment of severity, date of onset and resolution will be also documented. Abnormal laboratory results will only be documented as AEs if they are deemed to be of clinical significance.

An independent data monitoring committee (iDMC) will be responsible for the review of all unblinded safety data and will meet quarterly for the first year of the trial and every six months until the follow-up visit of the last patient. All iDMC meetings will be held without involvement of the trial team apart from the unblinded statistician. The trial management group (TMG) will also assess safety in a blinded manner on an ongoing basis during the trial.

Trial stopping criteria

For the first ten patients dosed, if a cumulative total of three serious adverse reactions (SARs) or severe adverse reactions (ARs) are observed the trial will be halted. This will trigger an unscheduled independent data monitoring committee meeting to review the unblinded trial data to date.

After the tenth patient has commenced dosing, a percentage basis (30%) of patients who experience pre-specified events (SARs or severe adverse reactions) will be used to halt the trial and trigger a review by the iDMC. Once the trial has halted, it will only resume after approval of a substantial amendment to the regulatory authorities that have approved the trial.

Patient and public involvement

Patient and public representatives were involved in the design of this study protocol. The NIHR Cambridge BRC Communications and PPI/E Department for PPI reviewed the patient facing documents and their feedback has been incorporated as much as possible.

ETHICS AND DISSEMINATION

For peer review only

The Health Research Authority and Health and Care Research Wales, UK (19/YH/0171), approved the study. All participants are required to read the patient information sheet in full and give written consent to participate in the trial. Annual reports will be submitted to the research ethics committee (REC) in accordance with national requirements. The results will be reported through peer-reviewed journals and conference presentations.

Acknowledgments

RS acknowledges funding from the Medical Research Council UK. TZ acknowledges funding from the British Heart Foundation. JT acknowledges funding from the Wellcome trust (211100/Z/18/Z). ZM acknowledges funding support from the British Heart Foundation (BHF) for his BHF Chair and from Cambridge Biomedical Research Centre. JHFR acknowledges funding support from HEFCE, the NIHR Cambridge Biomedical Research Centre, the British Heart Foundation, the EPSRC and the Wellcome Trust. JC acknowledges funding from The National Institute for Health Research (NIHR) Cambridge Biomedical Research Centre (BRC), which is a partnership between Cambridge University Hospitals NHS Foundation Trust and the University of Cambridge, funded by the National Institute for Health. Research (NIHR). This research was supported by the NIHR Cambridge Biomedical Research Centre (BRC-1215-20014). The views expressed are those of the author(s) and not necessarily those of the NIHR or the Department of Health and Social Care.
The authors acknowledge support from the Cambridge Clinical Research Centre, the Core Biochemistry Assay Laboratory and the Cambridge Clinical Trials Unit in the conduct, analysis and reporting of this clinical trial. The investigators also thank all the patients who participated in this trial, as well as the NHS Trusts and staff who have supported this trial.

Funding

This work was funded by the Medical Research Council, grant number MR/N028015/1 and the British Heart Foundation Cambridge Centre of Excellence (RCAG/521). Setup, running of the study and decision to publish results is independent of the funders.

Competing interests

None declared.

Patient consent

Not required.

Contributors

ZM, JC, JR, RS, TZ, SH, SB[3], JT, NB, HC were involved in the conception and design of the trial. JC is the principal investigator for this trial. RS, AH, VE, JH, TZ, PC were involved in data collection. HT co-ordinated the trial. VW, NB were involved in PETCT image acquisition. SB[5] and PK were involved immunology sample analysis. NJ performed transthoracic echocardiograms for this trial. RS, SB[3], JC will be involved in data analysis and finalising the statistical analysis plan. SB[3]

For peer review only

and JC will approve the trial Statistical Analysis Plan prior to database lock and analysis.

References

1. Abdolmaleki F, Hayat SMG, Bianconi V, et al. Atherosclerosis and immunity: a perspective. *Trends in cardiovascular medicine* 2019;29(6):363-71.
2. Ridker PM, Everett BM, Thuren T, et al. Antiinflammatory therapy with canakinumab for atherosclerotic disease. *New England journal of medicine* 2017;377(12):1119-31.
3. Libby P, Everett BM. Novel antiatherosclerotic therapies. *Arteriosclerosis, thrombosis, and vascular biology* 2019;39(4):538-45.
4. Kobiyama K, Saigusa R, Ley K. Vaccination against atherosclerosis. *Current opinion in immunology* 2019;59:15-24.
5. Ait-Oufella H, Salomon BL, Potteaux S, et al. Natural regulatory T cells control the development of atherosclerosis in mice. *Nature medicine* 2006;12(2):178.
6. Sharma M, Schlegel MP, Afonso MS, et al. Regulatory T cells license macrophage pro-resolving functions during atherosclerosis regression. *Circulation research* 2020;127(3):335-53.
7. Xiao J, Yu K, Li M, et al. The IL-2/Anti-IL-2 complex attenuates cardiac ischaemia-reperfusion injury through expansion of regulatory T cells. *Cellular Physiology and Biochemistry* 2017;44(5):1810-27.
8. Hofmann U, Beyersdorf N, Weirather J, et al. Activation of CD4+ T lymphocytes improves wound healing and survival after experimental myocardial infarction in mice. *Circulation* 2012;125(13):1652-63.
9. Matsumoto K, Ogawa M, Suzuki J-i, et al. Regulatory T lymphocytes attenuate myocardial infarction-induced ventricular remodeling in mice. *International heart journal* 2011;52(6):382-87.
10. Dobaczewski M, Xia Y, Bujak M, et al. CCR5 signaling suppresses inflammation and reduces adverse remodeling of the infarcted heart, mediating recruitment of regulatory T cells. *The American journal of pathology* 2010;176(5):2177-87.
11. Yu X, Newland SA, Zhao TX, et al. Innate lymphoid cells promote recovery of ventricular function after myocardial infarction. *Journal of the American College of Cardiology* 2021;78(11):1127-42.
12. Wigren M, Björkbacka H, Andersson L, et al. Low levels of circulating CD4+ FoxP3+ T cells are associated with an increased risk for development of myocardial infarction but not for stroke. *Arteriosclerosis, thrombosis, and vascular biology* 2012;32(8):2000-04.
13. Cheng X, Yu X, Ding Y-j, et al. The Th17/Treg imbalance in patients with acute coronary syndrome. *Clinical immunology* 2008;127(1):89-97.
14. Wolf D, Ley K. Immunity and inflammation in atherosclerosis. *Circulation research* 2019;124(2):315-27.
15. Han S-f, Liu P, Zhang W, et al. The opposite-direction modulation of CD4+ CD25+ Tregs and T helper 1 cells in acute coronary syndromes. *Clinical immunology* 2007;124(1):90-97.
16. Ammirati E, Cianflone D, Banfi M, et al. Circulating CD4+ CD25hiCD127lo regulatory T-Cell levels do not reflect the extent or severity of carotid and

For peer review only

coronary atherosclerosis. *Arteriosclerosis, thrombosis, and vascular biology* 2010;30(9):1832-41.
17. Klatzmann D, Abbas AK. The promise of low-dose interleukin-2 therapy for autoimmune and inflammatory diseases. *Nature Reviews Immunology* 2015;15(5):283-94.
18. Zhao TX, Newland SA, Mallat Z. 2019 ATVB plenary lecture: interleukin-2 therapy in cardiovascular disease: the potential to regulate innate and adaptive immunity. *Arteriosclerosis, thrombosis, and vascular biology* 2020;40(4):853-64.
19. Sakaguchi S, Sakaguchi N, Asano M, et al. Immunologic self-tolerance maintained by activated T cells expressing IL-2 receptor alpha-chains (CD25). Breakdown of a single mechanism of self-tolerance causes various autoimmune diseases. *The Journal of Immunology* 1995;155(3):1151-64.
20. Vang KB, Yang J, Mahmud SA, et al. IL-2,-7, and-15, but not thymic stromal lymphopoeitin, redundantly govern CD4+ Foxp3+ regulatory T cell development. *The Journal of Immunology* 2008;181(5):3285-90.
21. Humrich JY, von Spee-Mayer C, Siegert E, et al. Rapid induction of clinical remission by low-dose interleukin-2 in a patient with refractory SLE. *Annals of the rheumatic diseases* 2015;74(4):791-92.
22. Matsuoka K-i, Koreth J, Kim HT, et al. Low-dose interleukin-2 therapy restores regulatory T cell homeostasis in patients with chronic graft-versus-host disease. *Science translational medicine* 2013;5(179):179ra43-79ra43.
23. He J, Zhang X, Wei Y, et al. Low-dose interleukin-2 treatment selectively modulates CD4+ T cell subsets in patients with systemic lupus erythematosus. *Nature medicine* 2016;22(9):991.
24. Koreth J, Matsuoka K-i, Kim HT, et al. Interleukin-2 and regulatory T cells in graft-versus-host disease. *New England Journal of Medicine* 2011;365(22):2055-66.
25. Rosenzwajg M, Lorenzon R, Cacoub P, et al. Immunological and clinical effects of low-dose interleukin-2 across 11 autoimmune diseases in a single, open clinical trial. *Annals of the rheumatic diseases* 2019;78(2):209-17.
26. Zhao T, Sriranjan R, Tuong Z, et al. Regulatory T Cell Response to Low-Dose IL-2 in Ischemic Heart Disease. *NEJM evidence* 2021;1(1)
27. Cheng VY, Slomka PJ, Le Meunier L, et al. Coronary arterial 18F-FDG uptake by fusion of PET and coronary CT angiography at sites of percutaneous stenting for acute myocardial infarction and stable coronary artery disease. *Journal of Nuclear Medicine* 2012;53(4):575-83.
28. Tarkin JM, Joshi FR, Evans NR, et al. Detection of atherosclerotic inflammation by 68Ga-DOTATATE PET compared to [18F] FDG PET imaging. *Journal of the American College of Cardiology* 2017;69(14):1774-91.
29. Folco EJ, Sheikine Y, Rocha VZ, et al. Hypoxia but not inflammation augments glucose uptake in human macrophages: Implications for imaging atherosclerosis with 18fluorine-labeled 2-deoxy-D-glucose positron emission tomography. *Journal of the American College of Cardiology* 2011;58(6):603-14.

For peer review only

30. Ogawa M NS, Saito Y, Kosugi M, Magata Y. What can be seen by 18F-FDG PET in atherosclerosis imaging? The effect of foam cell formation on 18F-FDG uptake to macrophages in vitro. *J Nucl Med* 2012;53(1):55-8.
31. Tawakol A, Migrino RQ, Bashian GG, et al. In vivo 18F-fluorodeoxyglucose positron emission tomography imaging provides a noninvasive measure of carotid plaque inflammation in patients. *J Am Coll Cardiol* 2006;48(9):1818-24.
32. Rudd JH, Myers KS, Bansilal S, et al. Atherosclerosis inflammation imaging with 18F-FDG PET: carotid, iliac, and femoral uptake reproducibility, quantification methods, and recommendations. *Journal of Nuclear Medicine* 2008
33. Tarkin JM, Joshi FR, Rudd JH. PET imaging of inflammation in atherosclerosis. *Nat Rev Cardiol* 2014;11(8):443-57.
34. Fayad ZA, Mani V, Woodward M, et al. Safety and efficacy of dalcetrapib on atherosclerotic disease using novel non-invasive multimodality imaging (dal-PLAQUE): a randomised clinical trial. *Lancet* 2011;378(9802):1547-59.
35. Elkhawad M, Rudd JH, Sarov-Blat L, et al. Effects of p38 mitogen-activated protein kinase inhibition on vascular and systemic inflammation in patients with atherosclerosis. *JACC: Cardiovascular Imaging* 2012;5(9):911-22.
36. Mäki-Petäjä KM, Elkhawad M, Cheriyan J, et al. Anti-tumor necrosis factor-α therapy reduces aortic inflammation and stiffness in patients with rheumatoid arthritis. *Circulation* 2012:CIRCULATIONAHA. 112.120410.
37. Tahara N, Kai H, Ishibashi M, et al. Simvastatin attenuates plaque inflammation: evaluation by fluorodeoxyglucose positron emission tomography. *Journal of the American College of Cardiology* 2006;48(9):1825-31.
38. Figueroa AL, Abdelbaky A, Truong QA, et al. Measurement of arterial activity on routine FDG PET/CT images improves prediction of risk of future CV events. *JACC: Cardiovascular Imaging* 2013;6(12):1250-59.
39. Tawakol A, Singh P, Rudd JH, et al. Effect of treatment for 12 weeks with rilapladib, a lipoprotein-associated phospholipase A2 inhibitor, on arterial inflammation as assessed with 18F-fluorodeoxyglucose-positron emission tomography imaging. *Journal of the American College of Cardiology* 2014;63(1):86-88.
40. Bucerius J, Hyafil F, Verberne HJ, et al. Position paper of the Cardiovascular Committee of the European Association of Nuclear Medicine (EANM) on PET imaging of atherosclerosis. *European journal of nuclear medicine and molecular imaging* 2016;43(4):780-92.
41. O'Donoghue ML, Braunwald E, White HD, et al. Effect of darapladib on major coronary events after an acute coronary syndrome: the SOLID-TIMI 52 randomized clinical trial. *Jama* 2014;312(10):1006-15.
42. Schwartz GG, Olsson AG, Abt M, et al. Effects of dalcetrapib in patients with a recent acute coronary syndrome. *New England Journal of Medicine* 2012;367(22):2089-99.
43. Investigators S. Darapladib for preventing ischemic events in stable coronary heart disease. *New England Journal of Medicine* 2014;370(18):1702-11.

For peer review only

44. He J, Zhang X, Wei Y, et al. Low-dose interleukin-2 treatment selectively modulates CD4+ T cell subsets in patients with systemic lupus erythematosus. *Nature medicine* 2016;22(9):991-93.

Figure titles

Figure 1. Summary of trial design
Figure 2. IVORY trial visits – timeline and assessments
Figure 3. Phenotypic characterisation and antibodies used in fluorescence-activated cell sorting (FACS) analysis of T cell subsets
Figure 4. Analysis methods used to quantify vascular inflammation in the index vessel

For peer review only

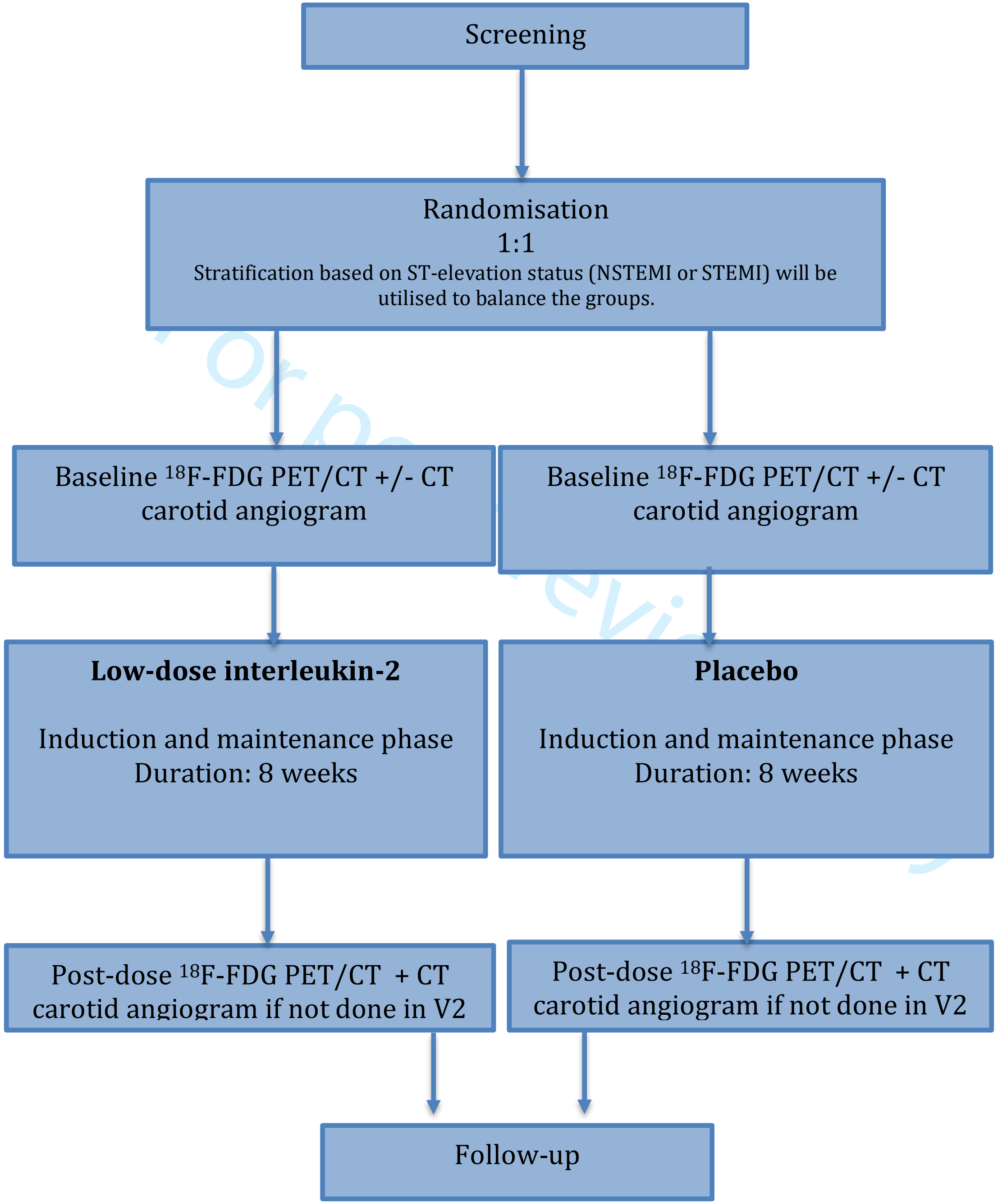

Figure 1:

Summary of Trial Design

Figure 2 : IVORY Trial timeline of visits and assessments

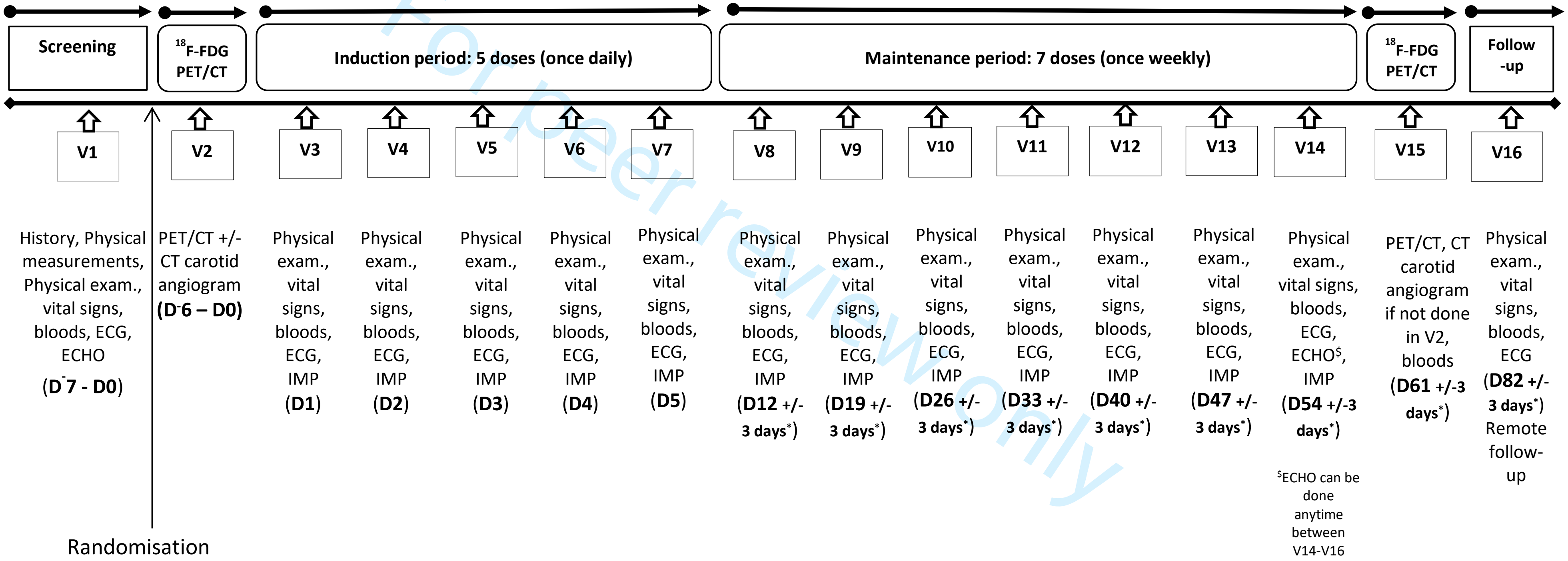

For peer review only - http://bmjopen.bmj.com/site/about/guidelines.xhtml

3a: Phenotypic characterization of T cell subsets by flow cytometry[44]

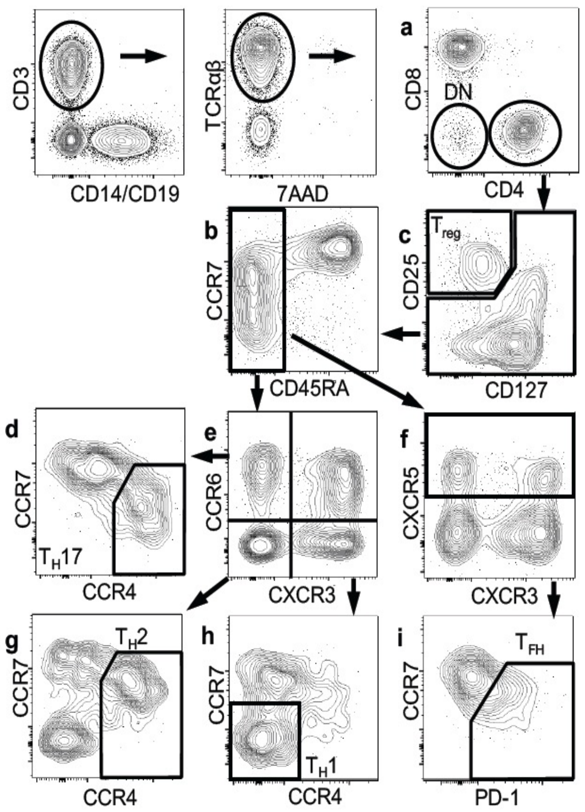

DN (CD4-CD8-)	CD4- CD8 -CD3+ TCRαβ+ CD14- CD19- 7AAD-
T_{reg}	CD25high CD127low CD4+ CD8 -CD3+ TCRαβ+ CD14- CD19- 7AAD-
T_H1	CXCR3+ CCR6- CCR4- CCR7low CD4+ CD8 -CD3+ TCRαβ+ CD14- CD19- 7AAD-
T_H2	CCR4+ CXCR3- CCR6- CCR7low CD4+ CD8 -CD3+ TCRαβ+ CD14- CD19- 7AAD-
T_H17	CCR4+ CCR6+ CXCR3- CCR7low CD4+ CD8 -CD3+ TCRαβ+ CD14- CD19- 7AAD-
T_{FH}	CXCR5+ PD-1+ CCR7low CD4+ CD8 -CD3+ TCRαβ+ CD14- CD19- 7AAD-

3b: Antibodies used in flow cytometry T cell subset analysis

Antibody	CD	BD Cat NO:
**CCR6**	CD196	**551773** BD Pharmingen™ PE Mouse Anti-Human
**CD25**	CD25	**564467** BD Horizon™ BB515 Mouse Anti-Human
**CCR4**	CD194	**566475** BD Pharmingen™ BB700 Mouse Anti-Human
**CCR7**	CD197	**557648** BD Pharmingen™ PE-Cy™7 Rat Anti-Human
**CXCR5**	CD185	**558113** BD Pharmingen™ Alexa Fluor® 647 Rat Anti-Human
**CD4**	CD4	**557922** Alexa Fluor® 700 Mouse Anti-Human
**CD45RA**	CD45RA	**560674** BD Pharmingen™ APC-H7 Mouse Anti-Human
**CXCR3**	CD183	**562558** BD Horizon™ BV421 Mouse Anti-Human
**CD3**	CD3	**563109** BV510 Mouse Anti-Human
**CD127 (IL-7Rα)**	CD127	**562662** BD Horizon™ BV605 Mouse Anti-Human
**CD8**	CD8	**563677** BD Horizon™ BV711 Mouse Anti-Human
**PD-1**	CD279	**563789** BD Horizon™ BV711 Mouse Anti-Human

Figure 4: Analysis Methods Used to Quantify Vascular Inflammation in the Index Vessel

A series of contiguous segments from the index vessel are illustrated. Atherosclerotic plaques in the vessel wall are indicated in yellow. the value within each segment represents the maximum tissue-to-background ratio (TBR) for that segment. Active segments were predefined as segments with TBR >1.6.[34]

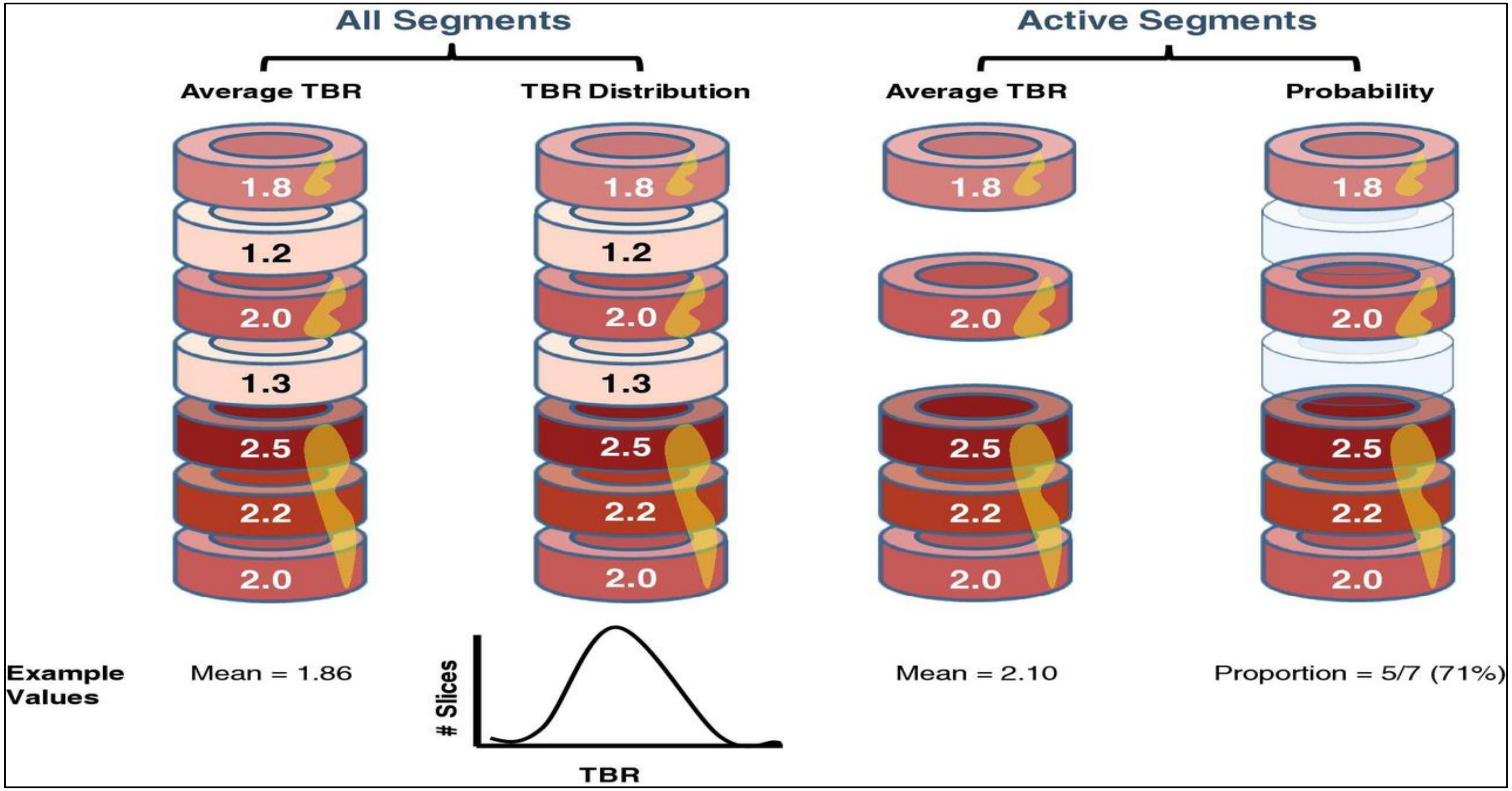

For peer review only

Supplementary Material

Supplementary Table 1 :
IVORY Inclusion and Exclusion Criteria

Inclusion Criteria

- Able to provide written informed consent to participate
- Aged between 18 and 85
- Current admission (on the screening visit) with an acute coronary syndrome - ST elevation myocardial infarction (STEMI), non-ST elevation myocardial infarction (NSTEMI), or unstable angina (UA) with symptoms suggestive of myocardial ischaemia lasting 10 minutes or longer with the patient at rest or with minimal effort

  AND EITHER

  i. elevated levels of troponin on admission

  OR

  ii. dynamic changes in ECG (new ST-T changes or T-wave inversion).

- Where applicable, to be included in the trial women must be

i) Postmenopausal (for the purposes of this trial, postmenopausal is defined as being amenorrhoeic for greater than 2 years with an appropriate clinical profile, e.g., age appropriate, history of vasomotor symptoms)

1. OR

ii) Have had a documented hysterectomy and/or bilateral oophorectomy or sterilised

1. OR

iii) Perimenopausal with a negative pregnancy test at screening (for the purposes of inclusion in this trial, perimenopausal is defined as women with an appropriate clinical profile, e.g., age appropriate, history of vasomotor symptoms, irregular periods). They will also have to comply with the use of contraception for the duration of the trial and undergo additional pregnancy tests during the trial.

- High sensitivity C-reactive protein of >2 mg/L at screening
- Willingness and possibility to start dosing within 14 days from initial date of admission to the primary hospital for ACS
- Able to comply with all trial mandated visits.

Exclusion Criteria

- Current presentation (at screening) with cardiogenic shock (systolic blood pressure <80 mmHg, unresponsive to fluids, or necessitating catecholamines).
- Current presentation with cardiac arrest
- Signs or symptoms of active infection requiring intravenous antibiotic treatment at screening
- History of malignancies requiring active treatment (however, patients with a history of treated localised basal or squamous cell skin cancer are not excluded from participation in this trial)
- History of solid organ transplantation or other bone marrow transplantation
- History of recurrent epileptic seizures in the previous 4 years; repetitive or difficult to control seizures, coma or toxic psychosis lasting >48 hours
- Uncontrolled hypotension (systolic blood pressure <80mmHg or diastolic blood pressure <50mmHg) OR uncontrolled hypertension (systolic blood pressure>180mmHg or diastolic blood pressure>120mmHg) at screening
- Average corrected QT interval (QTc) > 450msecs using Bazett's formula from average of triplicate ECGs or >480msecs if bundle branch block is present.
- Renal impairment defined as creatinine clearance [Cockcroft-Gault] <45ml/min at screening
- Liver dysfunction. Defined as alanine transaminase (ALT) >2 x upper limit of normal (ULN) at screening
- Evidence of cholestasis defined as elevated total bilirubin levels (TBL) > 1.5 x ULN and alkaline phosphatase (ALP) >1.5 x ULN at screening.
- Known hypothyroidism or hyperthyroidism
- Known autoimmune disease requiring active immunosuppressive treatment
- Any oral or intravenous immunosuppressive treatment including regular prednisolone, hydrocortisone or disease modifying drugs. [Inhaled or topical steroids are permissible]
- Patients on cytotoxic drugs and interferon-alpha
- Diabetics on oral hypoglycaemics/diet control with a haemoglobin A1c (HbA1c) diabetes control and complications assay (DCCT) > 8% OR HbA1c standardised to

international federation of clinical chemistry and laboratory medicine (IFCC) >64mmol/mol, at screening. Diabetics on insulin are excluded from the study.

- Contraindication to IL-2 treatment or hypersensitivity to IL-2 or to any of its excipients
- Participation in a previous research trial in the last 3 years which involved exposure to significant ionising radiation (i.e., cumulative research radiation dose >5mSv)
- Participation in a clinical trial where the patient has received a drug or new chemical entity within 30 days or 5 half-lives, or twice the duration of the biological effect of the drug (whichever is longer) prior to the first dose of trial medication.
- Any medical history or clinically relevant abnormality that is deemed by the principal investigator (PI)/delegate to make the patient ineligible for inclusion because of a safety concern
- Pregnant women or breast-feeding women
- Patients who are COVID-19 PCR positive at the time of screening
- Known severe allergy to the CT-contrast agents.

For peer review only

Patient Informed Consent form

Trial Title: Low-dose interleukin-2 for the reduction of vascular inflammation in Acute Coronary Syndromes (IVORY)

Principal Investigator: **Participant Number:** __________

If you agree with each sentence below, please initial the box **INITIALS**

1	I have read and understood the Participant Information Sheet version 3.0, dated 01 March 2021 for the above trial and I confirm that the trial procedures and information have been explained to me. I have had the opportunity to ask questions and I am satisfied with the answers and explanations provided.	
2	I understand that my participation in this trial is voluntary and that I am free to withdraw at any time, without giving a reason and without my medical care or legal rights being affected.	
3	I understand that personal information about me will be collected and used in accordance with this information sheet. This information will be kept in the strictest confidence and none of my personal data will be published.	
4	I understand that sections of my medical notes or information related directly to my participation in this trial may be looked at by responsible individuals from the sponsor, regulatory authorities and research personnel where it is relevant to my taking part in research and that they will keep my personal information confidential. I give permission for these individuals to have access to my records.	
5	I understand that my GP will be informed of my participation and any incidental clinical findings of relevance in this trial as well as information about the IVORY trial.	
6	I understand that my personal data might be transferred between the trial team at different trial sites in relation to my participation in this trial. I understand that any personal data will be sent using (secure/encrypted mail servers etc).	
7	I have read and understood the compensation arrangements for this trial as specified in the Participant Information Sheet.	
8	I understand that the doctors in charge of this trial may close the trial, or stop my participation in it at any time without my consent.	
9	I have read and understood my responsibilities for the trial including using appropriate contraception as listed in section 6.	
10	I understand that any data and samples already collected will still be used by the trial team if I withdraw/am withdrawn from the trial, unless I instruct the team to destroy them.	

OPTIONAL **YES NO**

11	I agree that in the event that there are any remaining unused samples at the end of the IVORY trial analysis these may be used in other approved research studies.		
12	I give my permission to be re-contacted in the future by this research team and other research teams to invite me to take part in new studies that may be of interest. I understand that this does not oblige me to take part in further research.		

For peer review only

I agree to participate in this trial:

______________________	______________________	__________
Name of patient	Signature	Date
______________________	______________________	__________
Name of person taking consent	Signature	Date

Time of Consent (24hr clock) ________:________

1 copy for the patient, 1 copy for the trial team, 1 copy to be retained in the hospital notes.

For peer review only

Reporting checklist for protocol of a clinical trial.

Based on the SPIRIT guidelines.

Instructions to authors

Complete this checklist by entering the page numbers from your manuscript where readers will find each of the items listed below.

Your article may not currently address all the items on the checklist. Please modify your text to include the missing information. If you are certain that an item does not apply, please write "n/a" and provide a short explanation.

Upload your completed checklist as an extra file when you submit to a journal.

In your methods section, say that you used the SPIRITreporting guidelines, and cite them as:

Chan A-W, Tetzlaff JM, Gøtzsche PC, Altman DG, Mann H, Berlin J, Dickersin K, Hróbjartsson A, Schulz KF, Parulekar WR, Krleža-Jerić K, Laupacis A, Moher D. SPIRIT 2013 Explanation and Elaboration: Guidance for protocols of clinical trials. BMJ. 2013;346:e7586

		Reporting Item	Page Number
**Administrative information**			
Title	#1	Descriptive title identifying the study design, population, interventions, and, if applicable, trial acronym	1
Trial registration	#2a	Trial identifier and registry name. If not yet registered, name of intended registry	2
Trial registration: data set	#2b	All items from the World Health Organization Trial Registration Data Set	1-12
Protocol version	#3	Date and version identifier	1
Funding	#4	Sources and types of financial, material, and other support	12
Roles and responsibilities: contributorship	#5a	Names, affiliations, and roles of protocol contributors	1,12

For peer review only

Roles and responsibilities: sponsor contact information	#5b	Name and contact information for the trial sponsor	5,6,12
Roles and responsibilities: sponsor and funder	#5c	Role of study sponsor and funders, if any, in study design; collection, management, analysis, and interpretation of data; writing of the report; and the decision to submit the report for publication, including whether they will have ultimate authority over any of these activities	5,12
Roles and responsibilities: committees	#5d	Composition, roles, and responsibilities of the coordinating centre, steering committee, endpoint adjudication committee, data management team, and other individuals or groups overseeing the trial, if applicable (see Item 21a for data monitoring committee)	11
**Introduction**			
Background and rationale	#6a	Description of research question and justification for undertaking the trial, including summary of relevant studies (published and unpublished) examining benefits and harms for each intervention	3,4
Background and rationale: choice of comparators	#6b	Explanation for choice of comparators	3,4
Objectives	#7	Specific objectives or hypotheses	3,4,9
Trial design	#8	Description of trial design including type of trial (eg, parallel group, crossover, factorial, single group), allocation ratio, and framework (eg, superiority, equivalence, non-inferiority, exploratory)	7
**Methods: Participants, interventions, and outcomes**			
Study setting	#9	Description of study settings (eg, community clinic, academic hospital) and list of countries where data will be collected. Reference to where list of study sites can be obtained	5

For peer review only

Eligibility criteria	#10	Inclusion and exclusion criteria for participants. If applicable, eligibility criteria for study centres and individuals who will perform the interventions (eg, surgeons, psychotherapists)	5
Interventions: description	#11a	Interventions for each group with sufficient detail to allow replication, including how and when they will be administered	6-9
Interventions: modifications	#11b	Criteria for discontinuing or modifying allocated interventions for a given trial participant (eg, drug dose change in response to harms, participant request, or improving / worsening disease)	7
Interventions: adherance	#11c	Strategies to improve adherence to intervention protocols, and any procedures for monitoring adherence (eg, drug tablet return; laboratory tests)	6-9
Interventions: concomitant care	#11d	Relevant concomitant care and interventions that are permitted or prohibited during the trial	6-9
Outcomes	#12	Primary, secondary, and other outcomes, including the specific measurement variable (eg, systolic blood pressure), analysis metric (eg, change from baseline, final value, time to event), method of aggregation (eg, median, proportion), and time point for each outcome. Explanation of the clinical relevance of chosen efficacy and harm outcomes is strongly recommended	9-11
Participant timeline	#13	Time schedule of enrolment, interventions (including any run-ins and washouts), assessments, and visits for participants. A schematic diagram is highly recommended (see Figure)	6-9
Sample size	#14	Estimated number of participants needed to achieve study objectives and how it was determined, including clinical and statistical assumptions supporting any sample size calculations	5-6
Recruitment	#15	Strategies for achieving adequate participant enrolment to reach target sample size	5-7
**Methods: Assignment of interventions (for controlled trials)**			
Allocation: sequence generation	#16a	Method of generating the allocation sequence (eg, computer-generated random numbers), and list of any factors for stratification. To reduce predictability of a random sequence, details of any planned restriction (eg, blocking) should be	6

For peer review only

		provided in a separate document that is unavailable to those who enrol participants or assign interventions	
Allocation concealment mechanism	#16b	Mechanism of implementing the allocation sequence (eg, central telephone; sequentially numbered, opaque, sealed envelopes), describing any steps to conceal the sequence until interventions are assigned	6
Allocation: implementation	#16c	Who will generate the allocation sequence, who will enrol participants, and who will assign participants to interventions	6
Blinding (masking)	#17a	Who will be blinded after assignment to interventions (eg, trial participants, care providers, outcome assessors, data analysts), and how	6-7
Blinding (masking): emergency unblinding	#17b	If blinded, circumstances under which unblinding is permissible, and procedure for revealing a participant's allocated intervention during the trial	7
**Methods: Data collection, management, and analysis**			
Data collection plan	#18a	Plans for assessment and collection of outcome, baseline, and other trial data, including any related processes to promote data quality (eg, duplicate measurements, training of assessors) and a description of study instruments (eg, questionnaires, laboratory tests) along with their reliability and validity, if known. Reference to where data collection forms can be found, if not in the protocol	6
Data collection plan: retention	#18b	Plans to promote participant retention and complete follow-up, including list of any outcome data to be collected for participants who discontinue or deviate from intervention protocols	6
Data management	#19	Plans for data entry, coding, security, and storage, including any related processes to promote data quality (eg, double data entry; range checks for data values). Reference to where details of data management procedures can be found, if not in the protocol	6
Statistics: outcomes	#20a	Statistical methods for analysing primary and secondary outcomes. Reference to where other details of the statistical analysis plan can be found, if not in the protocol	9-11

For peer review only

Statistics: additional analyses	#20b	Methods for any additional analyses (eg, subgroup and adjusted analyses)	9-11
Statistics: analysis population and missing data	#20c	Definition of analysis population relating to protocol non-adherence (eg, as randomised analysis), and any statistical methods to handle missing data (eg, multiple imputation)	9-11
**Methods: Monitoring**			
Data monitoring: formal committee	#21a	Composition of data monitoring committee (DMC); summary of its role and reporting structure; statement of whether it is independent from the sponsor and competing interests; and reference to where further details about its charter can be found, if not in the protocol. Alternatively, an explanation of why a DMC is not needed	11
Data monitoring: interim analysis	#21b	Description of any interim analyses and stopping guidelines, including who will have access to these interim results and make the final decision to terminate the trial	11
Harms	#22	Plans for collecting, assessing, reporting, and managing solicited and spontaneously reported adverse events and other unintended effects of trial interventions or trial conduct	11
Auditing	#23	Frequency and procedures for auditing trial conduct, if any, and whether the process will be independent from investigators and the sponsor	5-6
**Ethics and dissemination**			
Research ethics approval	#24	Plans for seeking research ethics committee / institutional review board (REC / IRB) approval	1
Protocol amendments	#25	Plans for communicating important protocol modifications (eg, changes to eligibility criteria, outcomes, analyses) to relevant parties (eg, investigators, REC / IRBs, trial participants, trial registries, journals, regulators)	4,11
Consent or assent	#26a	Who will obtain informed consent or assent from potential trial participants or authorised surrogates, and how (see Item 32)	6

For peer review only

Consent or assent: ancillary studies	#26b	Additional consent provisions for collection and use of participant data and biological specimens in ancillary studies, if applicable	6
Confidentiality	#27	How personal information about potential and enrolled participants will be collected, shared, and maintained in order to protect confidentiality before, during, and after the trial	6
Declaration of interests	#28	Financial and other competing interests for principal investigators for the overall trial and each study site	11
Data access	#29	Statement of who will have access to the final trial dataset, and disclosure of contractual agreements that limit such access for investigators	1,11
Ancillary and post trial care	#30	Provisions, if any, for ancillary and post-trial care, and for compensation to those who suffer harm from trial participation	6
Dissemination policy: trial results	#31a	Plans for investigators and sponsor to communicate trial results to participants, healthcare professionals, the public, and other relevant groups (eg, via publication, reporting in results databases, or other data sharing arrangements), including any publication restrictions	1
Dissemination policy: authorship	#31b	Authorship eligibility guidelines and any intended use of professional writers	11
Dissemination policy: reproducible research	#31c	Plans, if any, for granting public access to the full protocol, participant-level dataset, and statistical code	1,11
**Appendices**			
Informed consent materials	#32	Model consent form and other related documentation given to participants and authorised surrogates	6
Biological specimens	#33	Plans for collection, laboratory evaluation, and storage of biological specimens for genetic or molecular analysis in the current trial and for future use in ancillary studies, if applicable	6

The SPIRIT Explanation and Elaboration paper is distributed under the terms of the Creative Commons Attribution License CC-BY-NC. This checklist was completed on 02. March 2022 using https://www.goodreports.org/, a tool made by the EQUATOR Network in collaboration with Penelope.ai

For peer review only
