# Supplementary material for: Low-dose interleukin 2 for the reduction of vascular inflammation in acute coronary syndromes (IVORY): protocol and study rationale for a randomised, double-blind, placebo-controlled, phase II clinical trial
Source: BMJ Open. 2022 Oct 7;12(10):e062602. doi: 10.1136/bmjopen-2022-062602 (PMC9558794; doi:10.1136/bmjopen-2022-062602)
Supplement: Reviewer comments [file bmjopen-2022-062602.reviewer_comments.pdf]

PEER REVIEW HISTORY

BMJ Open publishes all reviews undertaken for accepted manuscripts. Reviewers are asked to complete a checklist review form (**http://bmjopen.bmj.com/site/about/resources/checklist.pdf**) and are provided with free text boxes to elaborate on their assessment. These free text comments are reproduced below.

ARTICLE DETAILS

**TITLE (PROVISIONAL)**	Low-dose interleukin-2 for the reduction of vascular inflammation in acute coronary syndromes (IVORY): protocol and study rationale for a randomised, double-blind, placebo-controlled, phase II clinical trial
**AUTHORS**	Sriranjan, Rouchelle; Zhao, Tian; Tarkin, Jason; Hubsch, Annette; Helmy, Joanna; Vamvaka, Evangelia; Jalaludeen, Navazh; Bond, Simon; Hoole, Stephen; Knott, Philip; Buckenham, Samantha; Warnes, Victoria; Bird, Nick; Cheow, Heok; Templin, Heike; Cacciottolo, Paul; Rudd, James; Mallat, Ziad; Cheriyan, Joseph

This article was not externally reviewed at BMJ Open. Protocols that have been independently assessed prior to submission to BMJ Open are usually fast-tracked to publication on the grounds that further substantial changes will not be possible. This independent assessment will usually be external, independent review for both a substantial grant award from a non-commercial or government funder and ethics approval.
